# **Mutant*Gossypium universal stress protein-2*** (***GUSP-2***) gene confers resistance to various abiotic stresses in ***E. coli**** BL-*21 **and *CIM-496-Gossypium hirsutum***

**DOI:** 10.1038/s41598-021-99900-x

**Published:** 2021-10-14

**Authors:** Muhammad Nadeem Hafeez, Mohsin Ahmad Khan, Bilal Sarwar, Sameera Hassan, Qurban Ali, Tayyab Husnain, Bushra Rashid

**Affiliations:** 1 Centre of Excellence in Molecular Biology, University of the Punjab Lahore, Lahore, Pakistan; 2 Drug Research Program, Division of Pharmaceutical Chemistry and Technology, Faculty of Pharmacy, University of Helsinki, Helsinki, Finland; 3 School of PhD Program in Cellular and Molecular Biotechnology, University of Teramo, Teramo, Italy; 4 Department of Pharmacy, University of Chieti – Pescara “G. d’Annunzio”, Chieti, Italy; 5 Institute of Molecular Biology and Biotechnology, University of Lahore, Lahore, Pakistan

**Keywords:** Expression systems, Plant biotechnology

## Abstract

*Gossypium arboreum* is considered a rich source of stress-responsive genes and the EST database revealed that most of its genes are uncharacterized. The full-length *Gossypium* universal stress protein-2 (GUSP-2) gene (510 bp) was cloned in *E. coli* and *Gossypium hirsutum*, characterized and point mutated at three positions, 352–354, Lysine to proline (M1-usp-2) & 214–216, aspartic acid to serine (M2-usp-2) & 145–147, Lysine to Threonine (M3-usp-2) to study its role in abiotic stress tolerance. It was found that heterologous expression of one mutant (M1-usp-2) provided enhanced tolerance against salt and osmotic stresses, recombinant cells have higher growth up to 10-5dilution in spot assay as compared to cells expressing W-usp-2 (wild type GUSP-2), M2-usp-2 and M3-usp-2 genes. M1-usp-2 gene transcript profiling exhibited significant expression (8.7 fold) in CIM-496-*Gossypium hirsutum* transgenic plants and enhance drought tolerance. However, little tolerance against heat and cold stresses in bacterial cells was observed. The results from our study concluded that the activity of GUSP-2 was enhanced in M1-usp-2 but wipe out in M2-usp-2 and M3-usp-2 response remained almost parallel to W-usp-2. Further, it was predicted through in silico analysis that M1-usp-2, W-usp-2 and M3-usp-2 may be directly involved in stress tolerance or function as a signaling molecule to activate the stress adaptive mechanism. However, further investigation will be required to ascertain its role in the adaptive mechanism of stress tolerance.

## Introduction

Abiotic stresses are major threat to environment and agriculture. Salinity, heat cold and drought are the abiotic environmental factors that adversely affect growth, limit productivity and geographic distribution of plants^1^. Plants are defending themselves by initiating diverse set of metabolic activities against abiotic stresses^2^. At cellular level numerous genes are involved, which are responsible to initiate defense mechanism of plants^3^. Cotton productivity is highly vulnerable to abiotic factors especially drought and salinity^4,5^. Its contribution to our national economy is significant because it provides raw material to our local textile industry. Its share in GDP is 1.6% and its value addition in agriculture is 7.8%^6^. Erratic rain fall, irregular irrigation and uncontrolled usage of ground water make cotton more defenseless and challenge us to formulate other measures to protect cotton against abiotic factors. Cotton (*Gossypium* spp.), is genetically diverse plant, it has four domesticated species such as *G. arboreum L*, *G. herbaceum L*, *G. barbadense L*. and *G. hirsutum L*. *G. arboreum* has many remarkable benefits over *G. hirsutum*, and has significant resistance against biotic and abiotic stresses especially drought and salinity, which makes it valuable gene pool for improving modern cotton cultivars^7–9^.

*Gossypium Universal stress Protein-2* (*GUSP-2*) has been identified and cloned from water stressed leaves of *G. arboreum*^10,11^. Sequence analysis showed that *GUSP-2* is highly similar to bacterial MJ0577-type of adenosine-triphosphate-binding *USP* protein, which has been proposed to function as molecular switch in dehydration stress adaptation^12^. Nucleotide sequence of this gene showed 81% sequence similarity while it’s encoded protein share 77% amino acid homology. Protein has high percentage of similarity (17% to 61%) to the *USP*s from a variety of bacteria and plants. From now, it invites researchers to map out its role in stress tolerance mechanism.

*E. coli* contains six *USP* proteins^13^. It has been reported the presence of *USP*-genes in different organisms, where they are playing role in response to heat shock, metabolic control, DNA management and cold shock^14–18^. In addition, USP is a regulatory protein, so, its efficiency can also be increased if we could manipulate its interactions. The expression and cellular function of newly identified *GUSP-2* gene (mutated and wild type) were analyzed to ascertain its possible role in abiotic stress tolerance mechanism. The study was carried out to understand the major metabolic pathways in connection with abiotic tolerance, which will be helpful in providing direction for future metabolic engineering for abiotic-stress tolerance.

## Materials and methods

### Total RNA isolation from drought stressed leaves of Gossypium arboreum

Seeds of FDH-171 *Gossypium arboreum* obtained from Centre of Excellence in Molecular Biology, University of the Punjab Lahore which serve as source of experimental seed materials to students, and grown in green house at temperature around 30° ± 2 °C and at light intensity 250–300 µmol m^-2^ s^−1^. It have been confirmed that the experimental samples of plants, including the collection of plant material, complied with relevant institutional, national, and international guidelines and legislation with appropriate permissions from Centre of Excellence in Molecular Biology, University of the Punjab Lahore, Pakistan for collection of plant specimens. After 120d (days) the grown plants were subjected to drought stress by withdrawing water for 15d. Total RNA was isolated from leaves of drought stressed (15d) cotton by using the method explained by Jaakola et al.^19^, and reverse transcribed with oligo (dT) primer by using RevertAid TM H minus first strand cDNA synthesis kit (ThermoFisher, cat #1631). Primers were designed by using *SnapGene* software (https://www.snapgene.com/resources/ta-gc-cloning/) for *GUSP-2* gene amplification and cloning (Table S7, Supplementary data).

### TA-Ligation of GUSP-2 Gene in pET-30b

*GUSP-2* was amplified by using {Fd-BamHI-E-Y + Rv-EcoRI-E-Y} primer pair (Table 1) and desired fragments were eluted from the gel (ThermoFisher elusion kit cat #0691). Eluted sample was ligated into pCR2.1-TOPO vector (TA-Cloning kit cat# K2020-20) and then transformed in competent *TOP10 E. coli* cells (obtained from CEMB culture collection facility). The confirmed TA-clone named “*Tw-GP-2*” after restriction digestion (BamHI, cat # ERoo52) and (EcoRI, cat # 15,202–013) and Sanger sequencing confirmation.

**Table 1:** Transformation efficiency of *Gossypium hirsutum.*

Construct	No. of Embryos	Survival (2–4 weeks)	Survival (7–8 weeks)	Plants in soil	Transformation Efficiency (T.E %)
p*W-usp-2*	2000	832	154	16	0.8%
p*M1-usp-2*	2000	756	132	18	0.9%
p*M3-usp-2*	2000	887	109	19	0.95%

### In Silico analysis

#### Site directed mutagenesis of GUSP-2 gene

Three point mutations were identified in *GUSP-2* gene (510 bp) with computational analysis. The Sequence of *GUSP-2* gene was retrieved from NCBI (Accession # EU107767) and 3D model was predicted through SWISS-MODEL expasy tool (https://swissmodel.expasy.org/ .expasy.org/) and evaluated with RAMACHANDRAN plot (http://mordred.bioc.cam.ac.uk/~rapper/rampage.php). MOE software (https://www.chemcomp.com/Products.htm) was used for the identification of ATP-binding sites in template protein structure which interacted with ATP. For the alignment of sequences of both template (2gm3.A) and GUSP-2-protein CLC-Bio work bench tool was used. During alignment corresponding mismatched amino acids of GUSP-2 were analyzed. The residues which were matched with template were left and the mismatched amino acids in GUSP-2 were changed according to the template and three mutated GUSP-2 protein models were designed and named as M1-usp-2, M2-usp-2, M3-usp-2 accordingly.

#### In vivo incorporation of deduced mutations

Three identified point mutations were integrated into *Tw-GP-2* (Gene-Art-Site-Directed-Mutagenesis kit, cat # A13282). The positive transformants were confirmed with restriction digestion (enzymes used BamHI & EcoRI) and Sanger sequencing. The resultant mutant clones were named as *Tm1-GP-2*, *Tm2-GP-2* and *Tm3-GP-2* and mutated genes were named as *M1-usp-2*, *M2-usp-2* and *M3-usp-2* respectively. These mutated TA-clones were used to produce recombinant pET-30b (cat # 69,910-3, Merck Millipore) expression vector for *E. coli*. The confirmed clones of pet-30b vector were named *M1-CeS1*, *M2-CeS2, M3-CeS2*, *W-CeS* and were proceeded for transformation into *E. coli TOP10* and then into *E. coli* expression strains *BL-*21-wild type, *BL-*21-uspA mutant, *BL-*21-uspB mutant, *BL-*21-uspC mutant, *BL-*21-uspABC mutant (Table S8, Supplementary data). The colonies obtained were confirmed with PCR and restriction digestion.

#### Functional validation of wild type and mutated (*M1-usp-2*, *M2-usp-2, M2-usp-2* and *W-usp-2*) genes in *E. coli* under various abiotic stress conditions

Spot and liquid culture assays were carried out to ascertain the function of mutated (*M1-usp-2*, *M2-usp-2, M3-usp-2*) and wild type (*W-usp-2*) genes under various abiotic stress treatments (salt, drought, heat, cold). Wild type and mutant *E. coli BL-21* cells transformed with *M1-CeS1*, *M2-CeS2, M3-CeS3* and *W-CeS* constructs and with pET30b vector alone (negative control) were grown overnight in Luria–Bertani (LB) media. Next day overnight culture was 1:100 diluted and allowed to grow to an absorbance (OD600 nm) 0.6 and induced by adding 1 mM IPTG and incubating further at 37 °C up to 12 h under various abiotic stress conditions. For the spot assay^18^ the absorbance of culture was adjusted to 0.6 (OD600 nm) by diluting using broth. Different serial dilutions (10^–2^, 10^–3^, 10^–4^, 10^–5^) of the culture were made, spotted on LB plates supplemented with salt (800 mM NaCl) and PEG (8%). For cold stress, serially diluted culture were spotted on LB plates and placed at 4 °C. Spotted plates were removed from 4 °C after 10d and photos were taken after 12 h of incubation at 37 °C. For heat stress, after 2 h of IPTG induction 1 ml culture (OD600 nm 0.6) was added in 10 ml media and incubated at elevated temperatures (46 °C). Samples were removed after 8 h and spotted on LB plates with serial dilutions (10^–2^, 10^–3^, 10^–4^, 10^–5^). For liquid culture assay (salt, osmotic and heat stresses) 1 ml culture after 2 h of IPTG induction (OD600 nm 0.6) was added in 10 ml media containing salt (NaCl 800 mM), PEG (8%) and incubated at 37 °C (for heat stress at 48 °C), with shaking (180 rpm). The aliquots were removed after every 2 h up to 12 h and absorbance (OD600 nm) was measured. Abiotic stress (salt, osmotic, heat & cold) tolerance was determined with respect to control cultures (bacterial cells and vector control).

#### RNA isolation and semi quantitative RT-PCR

Five samples were taken from each stress treatment (heat, osmotic and salt), total RNA isolated (RNA isolation kit, cat # 7020). cDNA was synthesized with oligo (dT) primer by using RevertAid TM H minus first strand cDNA synthesis kit (ThermoFisher, cat #1631). This cDNA was used for fold expression of mutated and wild type *GUSP-2* through RT-PCR.

#### SDS-PAGE analysis

Samples were taken from induced and un-induced cultures for protein confirmation with SDS-PAGE. Briefly, cells were lysed by sonication at 50% level 6–7 pulses for 20 s each. To the lysed cells, 2.5 µl Triton X100 and 2.0 µl β-mercaptoethanol were added and centrifuged at 6000 rpm. The pellet was again dissolved in lysis buffer and incubated at 37 °C for 30 min. In 100 µl of supernatant, 18 µl of 6X SDS loading dye was added and placed in boiling water for 8 min, cool down on ice for 5 min and centrifuged at 14000 rpm for 15 min at 4 °C. The supernatant was analyzed by SDS-PAGE analysis.

##### Incorporation of two mutations in *GUSP-2* for pCAMBIA 1301b

After scrutinizing the mutated *GUSP-2* genes for their role under osmotic and salt stresses it was decided *M1-usp-2* & *M3-usp-2* mutant genes should be verified for osmotic stress tolerance enhancement in cotton. Selected point mutations were incorporated into *Tw-GP-2p* TA construct for CAMBIA1301 (cat # M1592, Marker Gene Technologies Inc). The confirmed mutated TA clones were named *Tm1-GP-2p* and *Tm3-GP-2p.*

**Figure 1:**
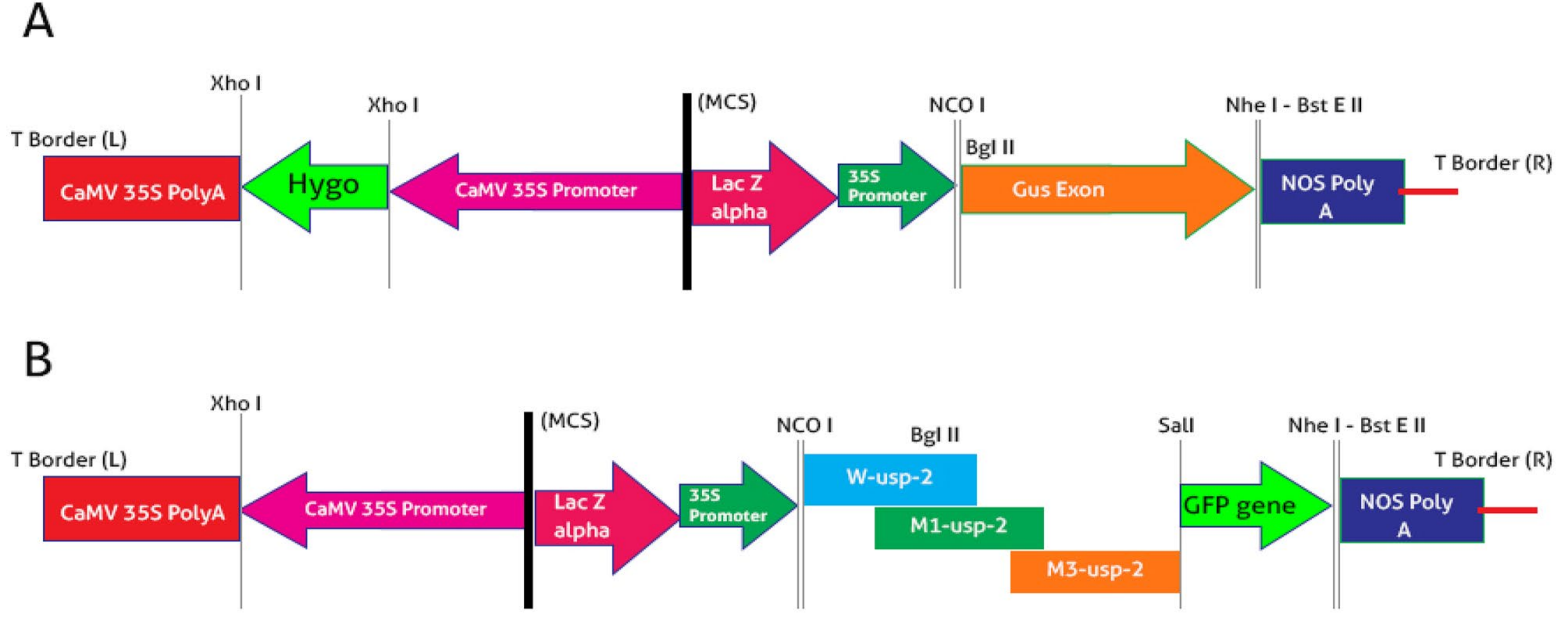
(**A**) pCAMBIA-1301 vector with hygromycin and (**B**) pCAMBIA-1301b vector without hygromycin and with wild type & mutated *GUSP-2* genes (cloned separately).

The size of pCAMBIA-1301 was reduced up to 10.8 kb from 11.8 kb by removing 1 kb fragment of hygromycin by using Xho1 site (Fig. 1A) and it was renamed pCEMBIA-1301b. The mutated and wild type *GUSP-2* genes were fused with *GFP* marker, pGWB5 vector was used for GFP (762 bp) amplification and TA cloning (Fd-SalI-GFP + Rv-BstEII-GFP primer pair).

Mutated and wild type TA constructs of *GUSP-2 (Tm1-GP-2p*, *Tm3-GP-2p* and *Tw-GP-2p*) and pCAMBIA-1301b vector (5 µl each) were double digested by using 1 µl of BglII (cat # ER0081) and 1 µl of SalI (cat # FR0641) and tango buffer (2 µl) having total volume 15 µl. Similarly, *GFP*-*TA* clone was double digested with SalI and BstEII. Then, three fragment ligation was conducted for *Tm1-GP-2-GFP*, *Tm3-GP-2-GFP* and *Tw-GP-2*-*GFP* fusion in pCAMBIA 1301b vector. GUS exon was replaced with genes *pM1-usp-2, pM3-usp-2*and *pW-usp-2* (Fig. 1A,B). Cloned pCAMBIA-1301b vector with mutated and wild type *GUSP-2* genes were confirmed through PCR, restriction digestion and Sanger sequencing. The resultant construct was named p*W-usp-2,* p*M1-usp-2* and p*M3-usp-2* and were transformed in *Agrobacterium tumefaciens-LBA-4404* for expression studies in local *G. hirsutum-CIM-496.*

#### *Agrobacterium* competent cells preparation

Single colony of *Agrobacterium tumefaciens-LBA-4404* was inoculated in 5 ml of YEP broth and the culture was incubated at 28 °C at 220 rpm for 72 h. Then, 1 ml culture was inoculated in120ml YEP broth (1:120 dilution) and again incubated at 28 °C at 220 rpm. When OD (600 nm) was reached at 0.6, then culture was centrifuged at 5000 rpm and pellet was washed with HEPES (pH7.0) 1 mM and then re-suspended in pre cold 10% glycerol.

#### Transformation of Gossypium hirsutum-CIM-496

BioRad electroporator was used for the transformation of confirmed constructs (p*GW-usp-2,* p*GM1-usp-2*, and p*GM3-usp-2*) into competent cells of *Agrobacterium- LBA-4404*. 2 µl of each plasmid construct (300 ng) was mixed with 120 µl of competent cells and proceed for electroporation and left at 28 °C incubation with 220 rpm shaking for 72 h. Then, the culture was spread onto the YEP-agar plates containing 50 µg/ml kanamycin. For initial confirmation of positive transformants, colony PCR was performed. The transformants were further confirmed with restriction digestion. *LBA 4404-Agrobacterium* transformed with recombinant plasmids was used to produce transgenic cotton plants.

#### Seed sterilization, and *Agrobacterium* culture treatment

Seeds of CIM-496 *G. hirsutum* were sterilized by adding few drops of Tween-20 in water by vigorous shaking and washed three times with autoclaved distilled water and were kept in the dark for germination at 30 °C overnight. Shoot cut apex method with some modifications was used for transformation as done by Gould et al.^20^. Total 2000 embryos were used in the transformation of each recombinant plasmid (pW-usp-2, pM1-usp-2& pM3-usp-2). After treatment with *Agrobacterium* embryos were shifted to MS medium and co-cultivated for 72 h. At this stage, antibiotics were not added to the media. The cultures were kept in growth room at 25 °C ± 2 °C and photoperiod of 16 h light and 8 h dark. The survival percentage of the embryos at different growth stages e.g. after co-cultivation, antibiotic selection, shoot and root formation was observed.

#### Selection on antibiotic medium and hardening of transgenic plants to soil

After three days (72 h) of co-cultivation, plantlets were shifted to selection medium i.e. MS plus kanamycin (100 mg/ml) and cefotaxime (250 μg/ml). Medium was also supplemented with different growth hormones i.e. Kinetin (1 mg/ml). Control plants were maintained on MS media with the 2.5 mg/L BAP + 0.1% (w/v) AC, rapid shoot growth occurred with kinetin supplemented media.. Plants were subcultured to fresh selection medium after every 10 days till 6–8 weeks and transformation efficiency was calculated.

Soil mixture (clay + sand + peat of 1:1:1) was prepared to shift the transformed plants from culture tubes to soil. Plants were kept in a growth room at 30 ºC + 2 °C for 16 h photoperiod in light intensity (250–300 µmol m^-2^ S^−1^). After 2–4 weeks plants were ready to shift to glass house.

#### Confirmation of transgenic plants

Transgenic plants were confirmed with PCR, and ELISA. Genomic DNA (GDNA) was isolated from leaves, shoots and roots of plants by following the procedure with modifications described by Dunwell^21^. Isolated GDNA was used for PCR amplification of *GUSP-2* gene by using primer pair (Fd-USP-full and Rv-USP-full). Total protein from root, stem and leaves was extracted according to the modified method described by Sambrook et al.^22^. For ELISA anti GFP monoclonal antibody (diluted 1:2500 in 1× PBS) was used to coat the wells of microtitre plate and incubated at 4 °C. Wells were washed with 1X PBS and 5% BSA and 100 μl of supernatant from each sample was added in wells and incubated at 25 °C. Washed with 1× PBS after 2 h of incubation and then secondary antibody (Sigma–Aldrich, USA, diluted 1:10,000) was added in wells and placed on shaker (30 rpm) for 2 h at 25 °C. Then NBT/BCIP substrate (Sigma–Aldrich, USA) was added in wells and left at 25 °C for 30–60 min. When color was developed then 1 N HCl was added to stop the reaction and the protein was quantified by using spectrophotometer at 450 nm_._

#### RT-qPCR expression analysis

Total RNA was isolated from different plant tissues (leaves, roots, stem) of control and drought stressed (15d) transgenic plants and cDNA was synthesized. Then spatial expression of transgene was conducted through RT-qPCR analysis. The expression analysis was carried on ABI 7500 real time PCR (Applied Biosystem, USA) by using SYBER green PCR master mix (Fermentas) with primer pairs (Fd-GAPDH + Rv- GAPDH & Fd-USP full + Rv-USP full). The GAPDH was used as a reference gene for data normalization. The relative gene expression analysis was done by using SDS 3.1 software (https://www.thermofisher.com/us/en/home/technical-resources/softwaredownloads/applied-biosystems-7500-real-time-pcr-system-for-human- identification.html) provided by ABI on the basis of CT values normalized with GAPDH.

#### Functional validation of genes in transgenic *CIM-496 G. hirsutum*

Functional analysis of wild type and mutant *GUSP-2* genes in transgenic plants was done by employing different physiological, morphological and biochemical parameters after inducing drought stress of 15d to the 120d old plants while control plants were watered regularly. Two types of control plants were also maintained. The transgenic (p*W-usp-2,* p*M1-usp-2,* p*M3-usp-2*) control plants of *CIM-496* were regularly watered and non-transgenic plants were used as negative control plants by withdrawing regular watering up to 15d.

### Morphological parameters

#### Plant height & Root length

Plant height before providing drought stress and after drought stress was measured. The initial height of the plants was measured from the soil surface to the apex before withholding water. Final height of the plants was measured at 15d of drought stress treatment. Five normal plants were selected at random and uprooted from each transgenic line and the length of their roots was measured from their tips to the base of hypocotyl and mean root length was expressed in centimeters.

### Physiological parameters

#### Relative water content

To determine relative water content (RWC), leaves were removed from selected plants as method described by Turner^23^ was followed. The (RWC) was determined using the following formula:

$${\text{Leaf}}\;{\text{Relative}}\;{\text{ Water}}\;{\text{ Content }}\left( {{\text{RWC}}} \right) = {\text{ FW}} - \frac{{{\text{DW}}}}{{{\text{TW}}}} - {\text{DW }} \times 100$$

#### Measurement of ion leakage (relative membrane permeability)

Leaves were removed from selected and were processed for the measurement of ion leakage from cellular membranes via conductivity measurement method. Detached leaves of drought-stressed and non-stressed plants were washed with deionized water and placed them on filter paper for air drying then placed in 25 ml of deionized water. Initial electrical conductivity (EC_0_) of deionized water was measured before the leaves to be soaked in it and final electrical conductivity (EC_1_) was determined after 24 h. Then leaves along with water were autoclaved and after autoclaving electrical conductivity was again measured (EC_2_). Ion leakage expressed in the form of percentage which was calculated according to the method described by Quisenberry^24^.

$$\% RMP = \frac{EC1 - EC0}{{EC2 - EC0}} \times 100$$

#### Photosynthesis

CO_2_ assimilation and transpiration rates were measured in the leaf with a handheld IRGA, gas exchange system (CI-340 Bioscientific Ltd., UK). The net photosynthesis rate by unit of leaf area (Pn, μmol CO_2_ m^−2^ s) was calculated.

### Biochemical parameters

#### Proline content

About 1 g leaf sample was crushed in 10 ml of 3% Sulfosalicylic acid and then the mixture was filtered through Whatman #1 filter paper. 2 ml of filtrate was mixed with 2 ml of acid ninhydrin in glass tubes and incubated for 1 h in boiling water bath. The reaction was terminated after 1 h by providing chilling temperature to the glass tubes. After cooling the reaction mixture, it was vigorously mixed with 4 ml toluene. Layers were formed within the reaction mixture when the glass tubes were allowed to settle at room temperature. The upper layer was separated and the intensity of red color, which indicating the presence of proline, was measured at 520_ nm_. The concentration of proline was estimated with the help of standard curve, which was drawn by using a known concentration of proline.

#### Confocal microscopy for GFP fluorescence

Transient expression of *GUSP-2* via *GFP* was determined in representative leaves through confocal microscopy. Transgenic and control leaves were taken, and slides were prepared for confocal imaging. Digital images were taken by confocal laser-scanning microscope (Zeiss LSM 510) equipped with argon laser. Argon laser was used for excitation of *GFP* at 488_ nm_ and emission wavelength 505_ nm_ was recorded as digital image.

### Statistical analysis

The results were statistically analyzed with software STATISTIX V 10.0 (Tallahassee, USA: https://statistix.informer.com). By using this statistical software factorial complete randomized design was applied on the data for analysis of variance (ANOVA). LSD (Lest Significant Difference) test was performed to access the significant differences among the transgenic and non-transgenic cotton plant.

## Results

### Isolation of GUSP-2 gene, TA-cloning

**Figure 2:**
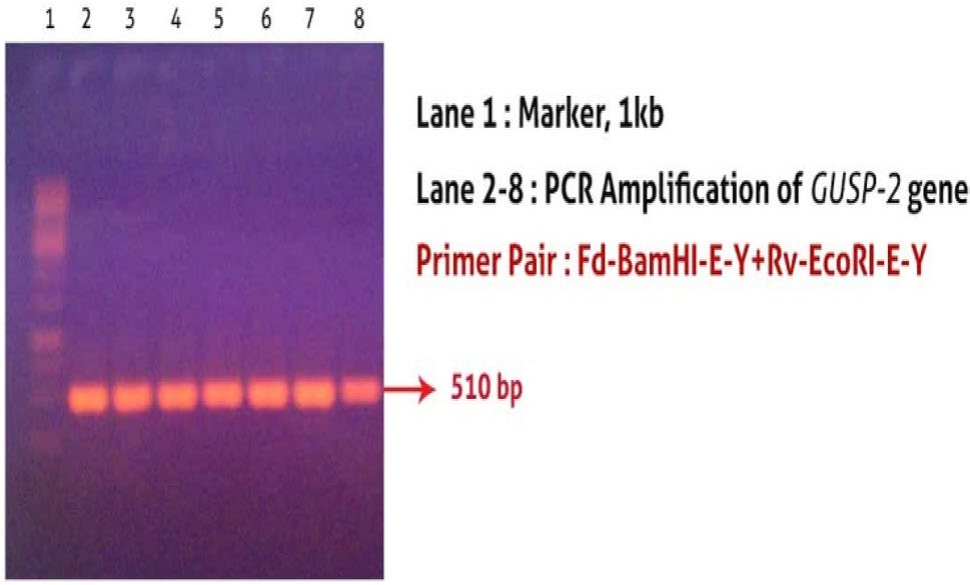
PCR amplification of *GUSP-2* gene.

Total RNA was extracted from leaves of FDH-171-*Gossypium arboreum* plants after drought stress of 15d and *GUSP-2* gene (Fig. 2) was amplified which showed clone number 3 and 5 generated desired fragments of 510 bp and 3.8 kb size (Fig. S1A,B, Supplementary data) and were proceed for Sanger sequencing (Fig. S2A,B, Supplementary data). TA-clone number 7 was proved correct and named *Tw-usp-2*.

### Site directed mutations of GUSP-2 gene

GUSP-2-protein sequence was aligned with 2gm3.A protein sequence, template protein which was used to predict GUSP-2-protein 3D-Model, to verify the mismatched residues in pocket region. The ATP-binding pocket residues of GUSP-2 were ensured while comparing with 2gm3.A, template protein. Two mismatched and one matched residue (Lysine 60, Aspartic acid 26 and Lysine 3) of GUSP-2 were selected for point mutations and were replaced with Proline, Serine and Threonine respectively by using MOE Bioinformatics tool. Three point mutations were separately created in GUSP-2-protein 3D-Model. (Fig. S3, Supplementary data).

### In vivo incorporation of mutations

Three mutations were separately incorporated in *Tw-usp-2*. Mutagenized plasmids (*Mp1, Mp2* & *Mp3*) were transformed into competent cells of DH5αT1R *E. coli* (Fig. S4A-C, Supplementary data). Positive transforment number 2, 6 from r*Mp1*, 2 from r*Mp2* and 5 from *rMp3* generated two fragments of 510 bp and 3.8 kb (Fig. S5A-C, Supplementary data) and were further confirmed with Sanger sequencing (Fig. S6A-C, Supplementary data). The confirmed clones were named *Tm1-GP-2*, *Tm2-GP-2 & Tm3-GP-2* respectively. TA constructs of mutants (*Tm1-GP-2, Tm2-GP-2* & *Tm3-GP-2*) were used for cloning pET-30b expression vector and transformation into wild type and mutated *E. coli* expression strains (Table S7, Supplementary data) for functional validation. Five colonies were picked randomly from each transforments (r*pE1, rpE2*, r*pE3 &* r*pW*) and screened via colony PCR (Fig. S7A,B, Supplementary data). Clone number 2 & 3 of r*pE1* (Fig. S8A, Supplementary data), 7, 9 & 11 of r*pE2* (Fig. S8B, Supplementary data), 2, 3, 4 & 6 of r*pE3* (Fig. S8B, Supplementary data) and 7, 8, 9 & 10 of r*pW* were positive. Positive transforment number 3 from r*pE1*, 9 from r*pE2,* 4 & 6 from r*pE3* and 8 & 9 from r*pEw* generated two fragments of 510 bp and 5.2 kb (Fig. S7A,B, Supplementary data) were confirmed positive and named *M1-CeS1*, *M2-CeS2, M3-CeS2* and *W-CeS* respectively.

### Functinal validation under various abiotic stress conditions

**Figure 3:**
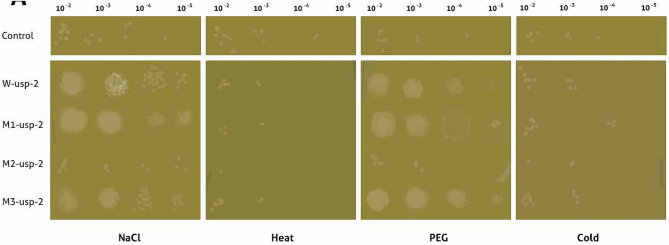
Spot assay of *E. coli-BL21-uspABC.* Transformed *BL21-usp-ABC* cells with wild type (*W-usp-2*) and mutant (*M1-usp-2*, *M2-usp-2, M3-usp-2*) genes were spotted on LB plates supplemented with salt (800 mM NaCl), PEG (8%). For heat stress, after 2 h of induction (IPTG) 1 ml culture was inoculated in 10 ml media (LB) and incubated at elevated temperatures (46 °C). Samples were removed after 8 h and spotted on respective plates. For cold stress, spotted plates were removed from 4 °C after 10d and photos were taken after incubation at 37 °C.

**Figure 4:**
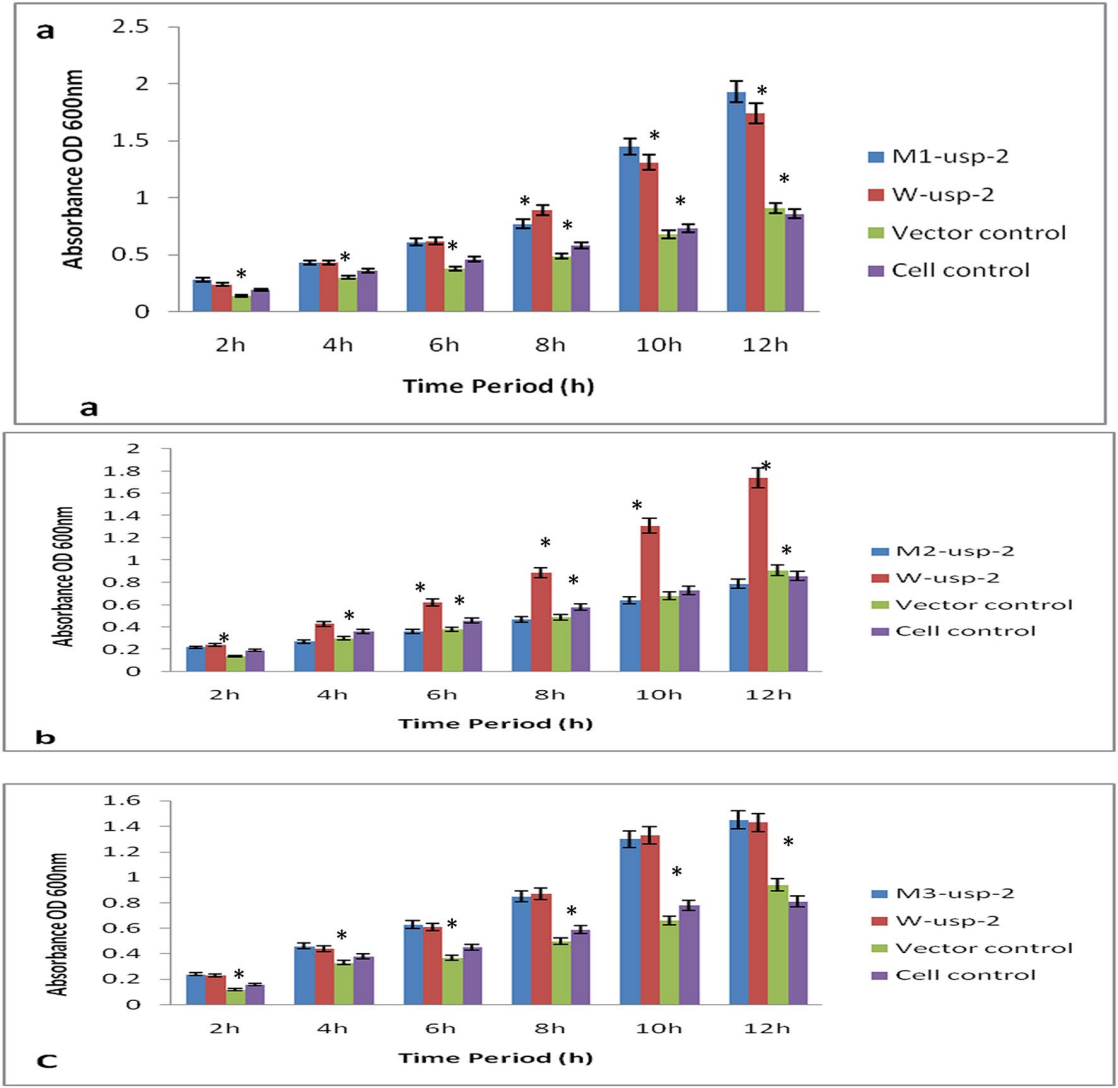
Comparison of growth under 800 mM (salt stress) in *E. coli BL-21-uspABC* mutant, expressing *W-usp-2*, *M1-usp-2, M2-usp-2* & *M3-usp-2* genes. (**a**) *E. coli BL-21-uspABC* mutant cells expressing *M1-usp-2* showed high tolerance then that of vector control, bacterial control cells and *W-usp-2* gene expressing cells. (**b**) *BL-21-uspABC* cells expressing *W-usp-2* gene are more tolerant as compared to *M2-usp-2* cloned cells of *BL-21-uspABC*. (**c**) growth rate of *BL-21-uspABC* cells transformed with *M3-usp-2* gene is almost similar to the growth rate of *BL-21-uspABC* cells expressing *W-usp-2* gene.

Under salt stress (NaCl 800 mM) mutant-1 (*M1-usp-2*) enhanced survival rate of *E. coli* cells as compared to cells transformed with *W-usp-2* and *M2-usp-2, M3-usp-2* genes (Fig. 3). Moreover, viability of *BL-21-uspA* mutant cells was found to be reduced than that of wild type *BL-21* cells under salt stress. Thus we assayed the sensitivity of all mutant strains for salt stress and found, the cells lacking all *USP* (*ABC*) genes were drastically more sensitive than that of *BL-21-uspA, BL-21-uspB, BL-21-uspC* and wild type *BL-21* cells, The comparative sensitivity of *BL-21-uspABC* mutant cells transformed with *M1-CeS1*, *M2-CeS2, M3-CeS2*, *W-CeS* constructs, *BL-21* control cells was shown in Fig. 4a-c. The OD (600 nm) after 12 h of growth was observed at 1.93 with *M1-usp-2* mutant gene (Fig. 4a). At the same time OD (600 nm) of *M2-usp-2* transformed cells was noted 0.79 for *E. coli BL-21-uspABC* (Fig. 4b) while OD of *W-usp-2* transformed cells were 1.74, which almost parallel to the OD of *M3-usp-2* transformed cells 1.45 (Fig. 4c).

**Figure 5:**
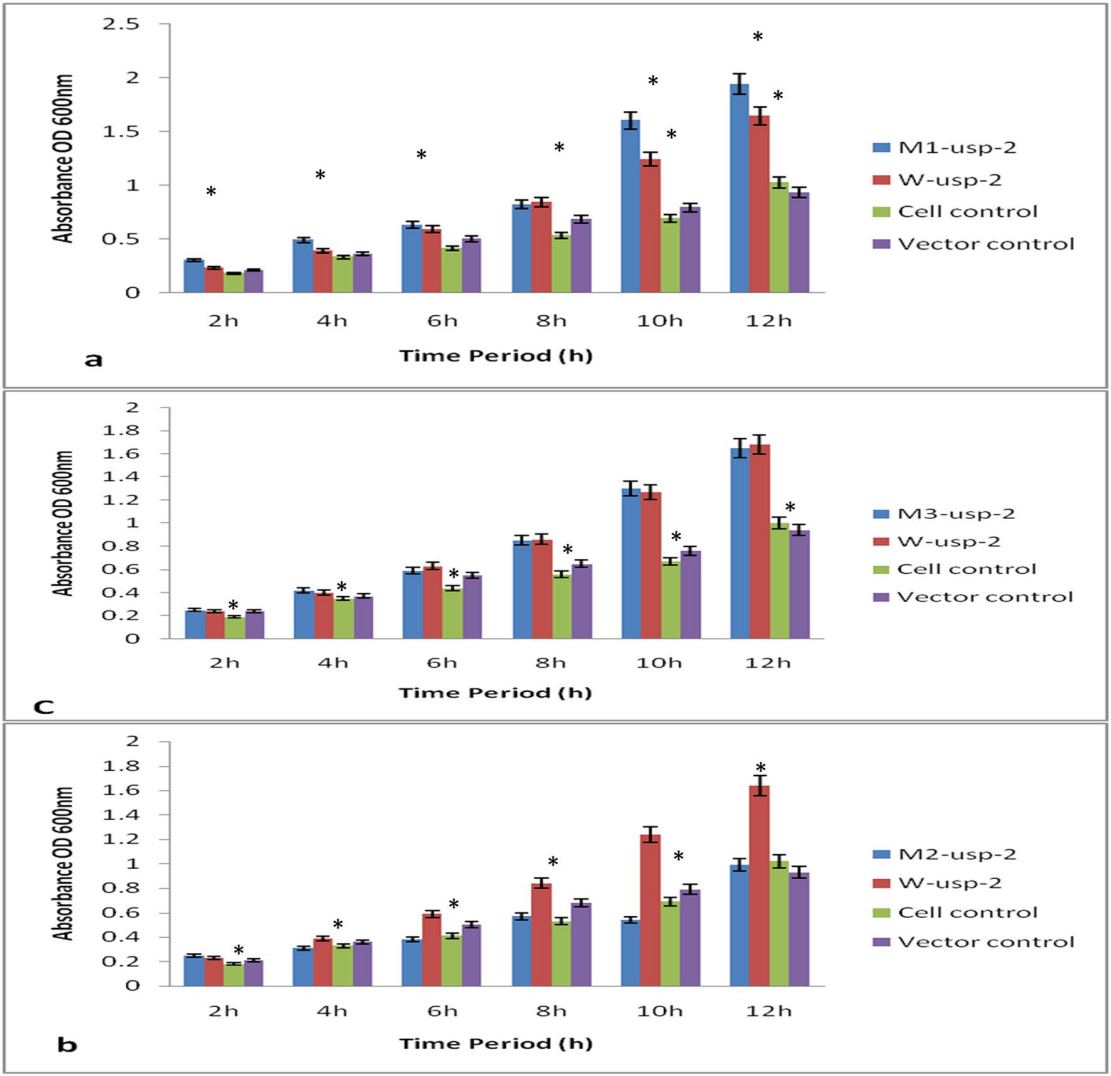
Osmotic stress tolerance in *E. coli BL-21*, (**a**) *E. coli BL-21 BL-21-uspABC* expressing *M1-usp-2* showed significant tolerance as compared to vector control, cell control and *W-usp-2* gene expressing cells, (**b**) *W-usp-2* gene expressing *BL-21-uspABC* cells are more tolerant as compared to *M2-usp-2* cloned cells of *E. coli*, (**c**) OD of *BL-21-uspABC* cells expressing *M3-usp-2* gene is almost similar to *BL-21-uspABC* mutant cells expressing *W-usp-2* gene.

Survival rate of bacterial cells under osmotic stress (PEG 8%) was observed stronger as compared to other abiotic stress conditions (heat & salt). Sensitivity of all mutant strains for PEG stress was analyzed and found, the cells lacking all *USP* (Fig. 3) genes were drastically more sensitive. Here we observed again that mutant-1 (*M1-usp-2*) has significant effect on cell survival as compared to mutant-2 (*M1-usp-2*), mutant-3 (*M3-usp-2*) and wild type (*W-usp-2*). The OD (600_ nm_) after 12 h of growth for *BL-21-uspABC* (*M1-usp-2* mutant) was 1.94 (Fig. 5a) and of *M2-usp-2* transformed cells was noted 0.99 for *BL-21-uspABC* (Fig. 5b) while OD of *W-usp-2* transformed cells were 1.64 which almost similar to the growth rate of bacterial cells transformed with *M3-usp-2* gene 1.65 (Fig. 5c).

**Figure 6:**
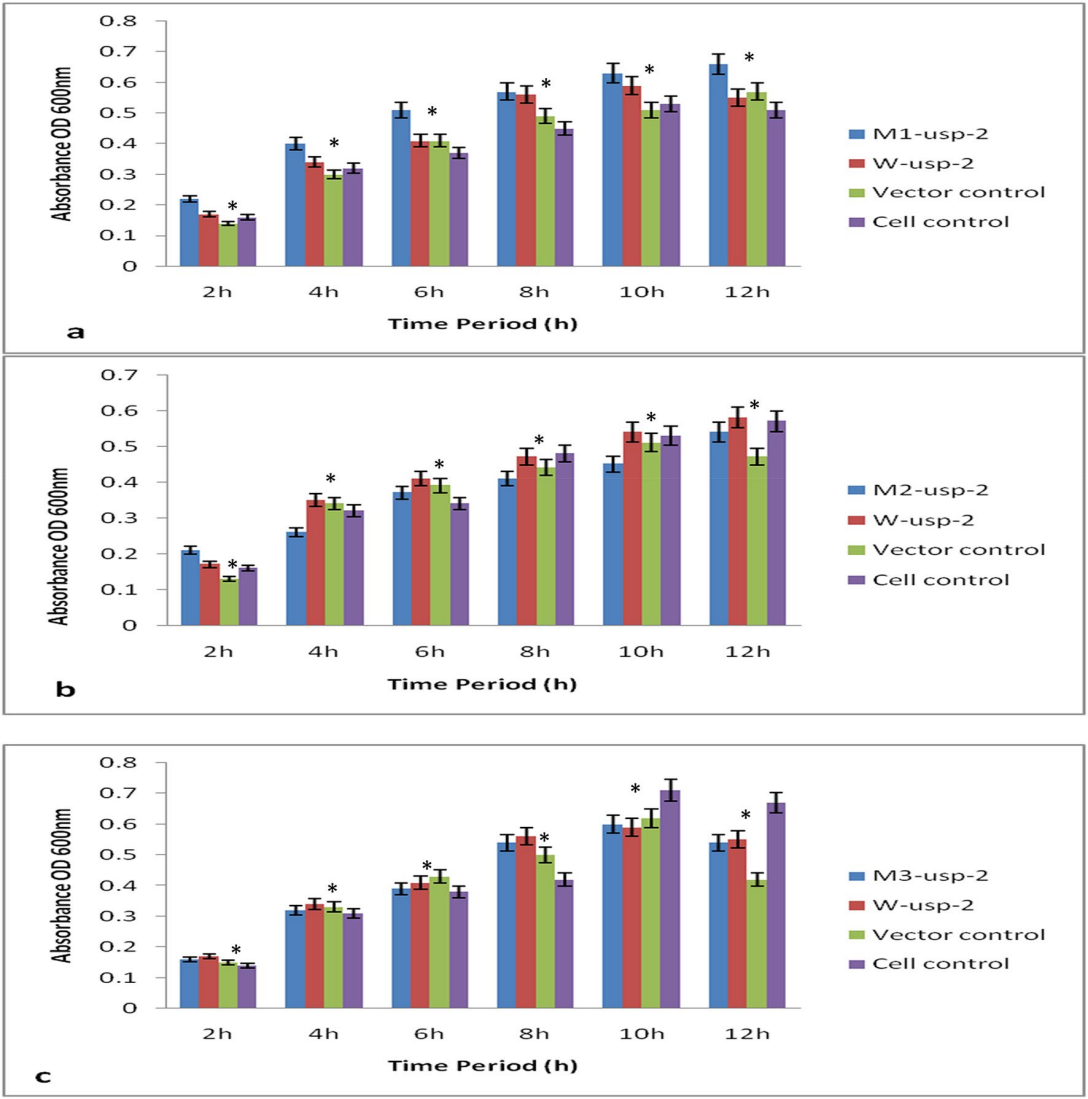
Comparison of Heat stress tolerance in *E. coli BL-21*. Both *E. coli BL-21-uspABC* (**a**–**c**) cells expressing *W-usp-2*, *M1-usp-2, M2-usp-2* and *M3-usp-2* showed slight tolerance as compared to control. (**b**) *W-usp-2* and *M3-usp-2* gene expressing *BL-21-uspABC* cells are modestly tolerant as compared to *M2-usp-2* cloned cells. It remained difficult to claim difference of survival rate among wild type and mutant clones.

Wild type (*W-usp-2*) and mutant (*M1-usp-2*) gene confer slight tolerance against heat stress in *E. coli* (Fig. 6). *BL-21-uspABC* cells expressing *M1-usp-2* gene showed modest tolerance (OD 0.76 after 12 h at 600 nm). Furthermore, *E. coli BL-21-usp-ABC* (OD 0.73 after 12 h at 600 nm) transformed with *W-usp-2* gene showed minute survival rate as compared to control cells. It was also observed, *E. coli* mutant cells transformed with *M3-usp-2* gene showed equivalent survival rate to *W-usp-2* expressing cells. However, mutant-2 (*M-2usp-2*) in cells (OD at 600 nm 0.54 after 12 h) failed to impart considerable resistance as compared to vector control and cell control (Fig. 6a-c).

### Relative expression level of under osmotic stress

**Figure 7:**
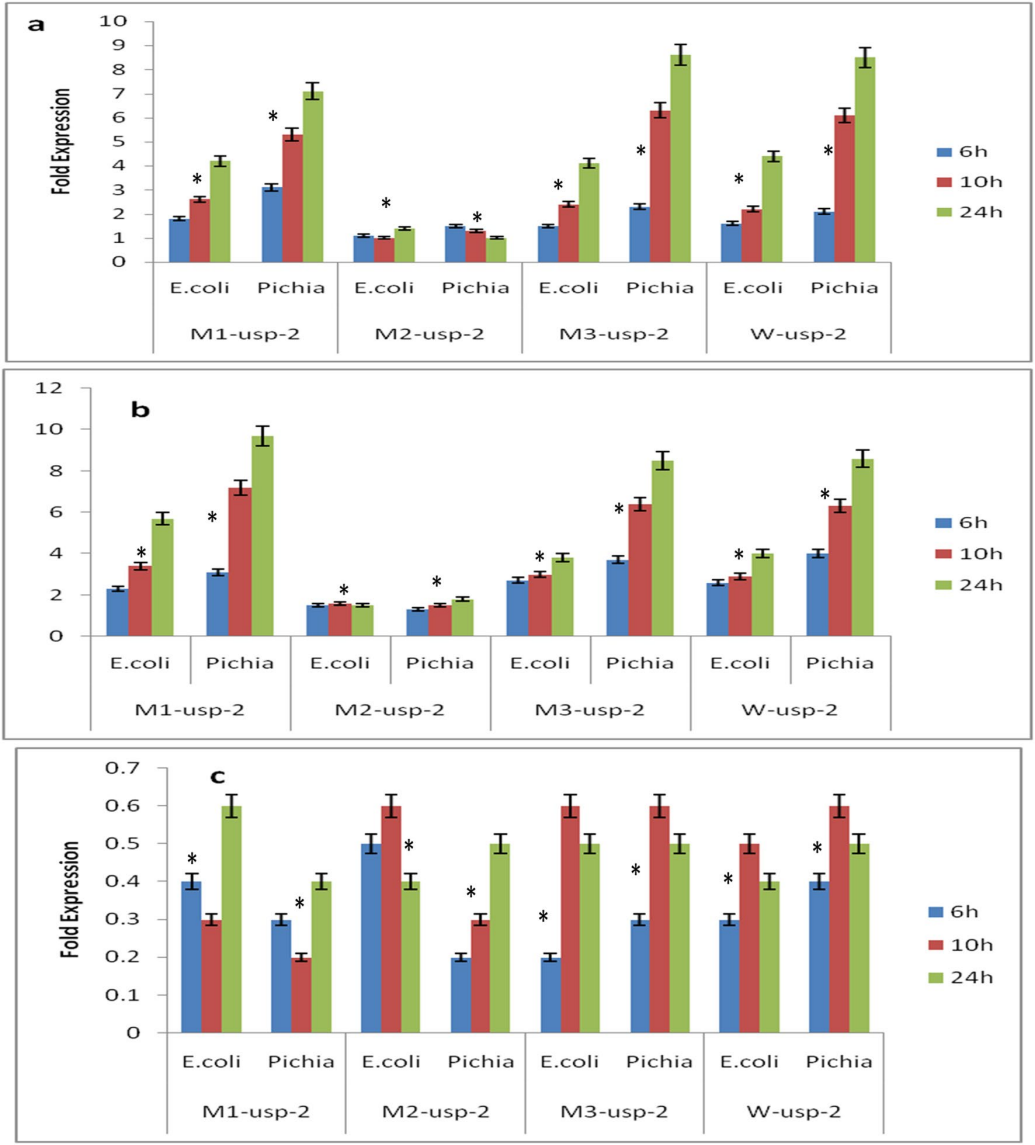
Relative expression level of mutant and wild type genes (*M1-usp-2*, *M2-usp-2, M3-usp-2* & *W-usp-2*) in *E. coli* and, data was analyzed by comparative CT method and presented as relative fold gene expression (2^−ΔΔC^T) with reference *rssA* gene. The graph indicates the mean ± SD (**a**) relative expression under NaCl 800 mM stress. Expression of *M1-usp-2* was significant in in *BL-21-uspABC cells* (4.2 fold). While *M2-usp-2 W-usp-2, M3-usp-2* genes have slight expression (4.4 & 4.1fold), (**b**) relative expression under PEG 8% stress. *M1-usp-2* relative expression was significant in *E. coli BL-21-uspABC* cells (5.7 fold). Again *M2-usp-2* gene expression was not notably different (4 & 3.8 fold) (**c**) relative expression under heat stress in *E. coli BL-21-uspABC* cells had not been observed at notable difference either because the death of cells at high temperature or because the wild type and mutant genes conferred no resistance against heat stress.

*M1-usp-2* expression under osmotic stress (8% PEG) in *BL-21-uspABC* cells was observed at uppermost level (5.7 folds) as compared to *M2-usp-2* (1.0 folds), *M3-usp-2* (3.8 folds) and *W-usp-2* (4.0 folds) (Fig. 7b). However, under heat (46 °C) stress all genes were poorly expressed (Fig. 7c) either because of cell death or genes don’t confer resistance against heat stress. Expression of *W-usp-2*, *M1-usp-2*, *M2-usp-2* & *M3-usp-2* genes were 0.4, 0.6, 0.4 & 0.5fold in *E. coli BL-21-uspABC*. Under salt stress expression level 4.4, 3.9, 4.1 & 4.2 of *W-usp-2*, *M1-usp-2*, *M2-usp-2* & *M3-usp-2* in *E. coli BL-21* (Fig. 7a).

### Incorporation of two mutations in GUSP-2 for plant expression vector

Two mutants (*M1-usp2* & *M3-usp-2*) and wild type genes of *GUSP-2* for the verification of osmotic stress tolerance enhancement in *CIM-496 G. hirsutum* were selected*. GUSP-2* was amplified product (Fd-BglII-P + Rv-SalI-P) and TA ligated in pCR2.1 vector. Five clonies (Fig. S9A,B, Supplementary data) were screened via clony PCR, colony number 1, 3, 4 & 5 were found positive. These positive transforments were confirmed with double digestion by using BglII and SalI enzymes (Fig. S10, Supplementary data). Clone number 1, 3 & 5 generated desired fragments of 510 bp and 3.8 kb. Similarly, *GFP* (762 bp) was TA cloned (Fig. S11, Supplementary data) and confirmed clone was named *T-GFp.* Lane1 maker 1 kb, Lane2-5 are positive PCR amplification. Positive transforments number 2, 3 & 4 were confirmed with double digestion (SalI and BstEII).

The selected mutants *M1-usp-2* & *M3-usp-2* were incorporated into *Tw-GP-2p* construct. The positive Mutagenized plasmids (*Gp1* &*Gp3*) were screened via colony PCR (Fig. S10, Supplementary data) and with restriction digestion (Fig. S12A,B, Supplementary data). The transformants were confirmed via Sanger sequencing and named *Tm1-GP-2p* and *Tm3-GP-2p.*

Hygromycin fragment (1 kb) was excised from pCAMBIA-1301 and was renamed pCEMBIA-1301b. Mutated and Wild type TA constructs of *GUSP-2 (Tm1-GP-2p*, *Tm3-GP-2p* and *Tw-GP-2p*) and pCEMBIA-1301b vector (5 µl each) and *T-GFp* were double digested. Then three fragments ligation was conducted for *Tm1-GP-2-GFP*, *Tm3-GP-2-GFP* and *Tw-GP-2*-*GFP* fusion in pCEMBIA 1301b vector (Fig. S13A-C, Supplementary data). Cloned pCAMBIA-1301b vectors were confirmed through colony PCR and restriction digestion and constructs were named p*W-usp-2,* p*M1-usp-2* and p*M3-usp-2* (Fig. S1A-C, Supplementary data) and were transformed in competent cells *Agrobacterium tumefaciens-LBA-4404*.

### Transformation of CIM-496 G. hirsutum

**Figure 8:**
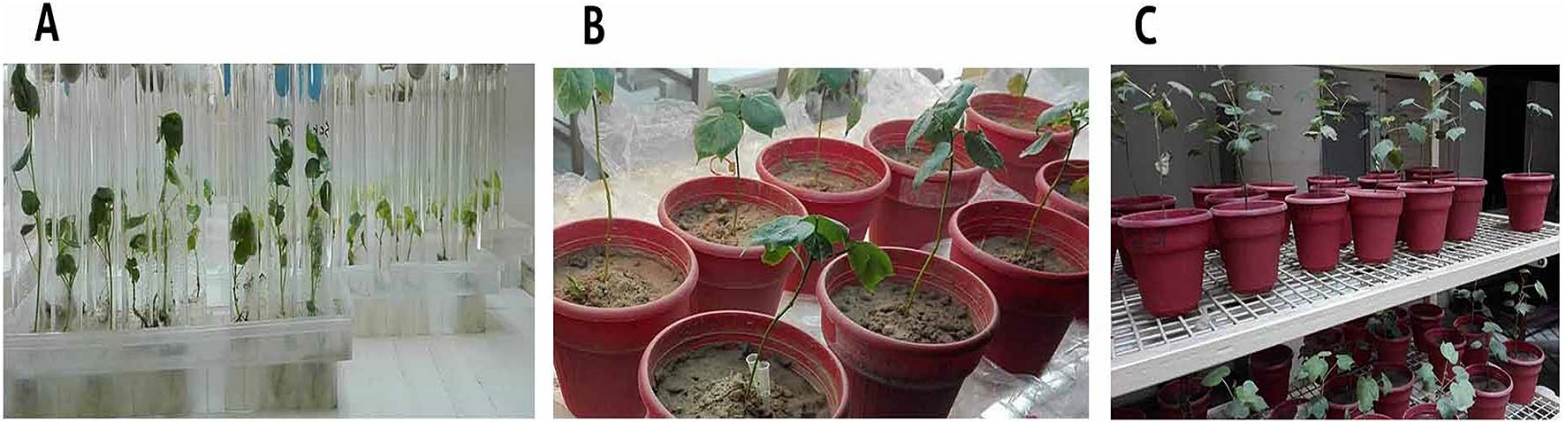
(**A**) Transgenic plants were growing in culture tubes, (**B**, **C**) Transgenic plants were shifted to pots.

The confirmed p*W-usp-2,* p*M1-usp-2* and p*M3-usp-2* clones were used to make transgenic *CIM-496 G. hirsutum*. After three weeks of growth on selection (kanamycin) medium, transgenic cotton plants were switched to shoot and root induction medium. After one and half month, healthy plants with prominent roots were shifted to pots containing loamy soil (Fig. 8A-C).

### Transformation efficiency

Total 6000 mature embryos were used in all the transformation experiments. After four weeks of selection on kanamycin (100 mg/ml), 154 plants of p*W-usp-2, 132* p*M1-usp-2* and 109 plants of p*M3-usp-2* were obtained. Only 16 plants of p*W-usp-2* out of 88, 11 plants of p*M1-usp-2* out of 91 and 9 plants of p*M3-usp2* out of 76 were successfully shifted to CEMB-greenhouse. The overall transformation efficiency remains 0.83% (Table 1).

### Confirmation of transgenic plants of *CIM-496 G. hirsutum*

**Figure 9:**
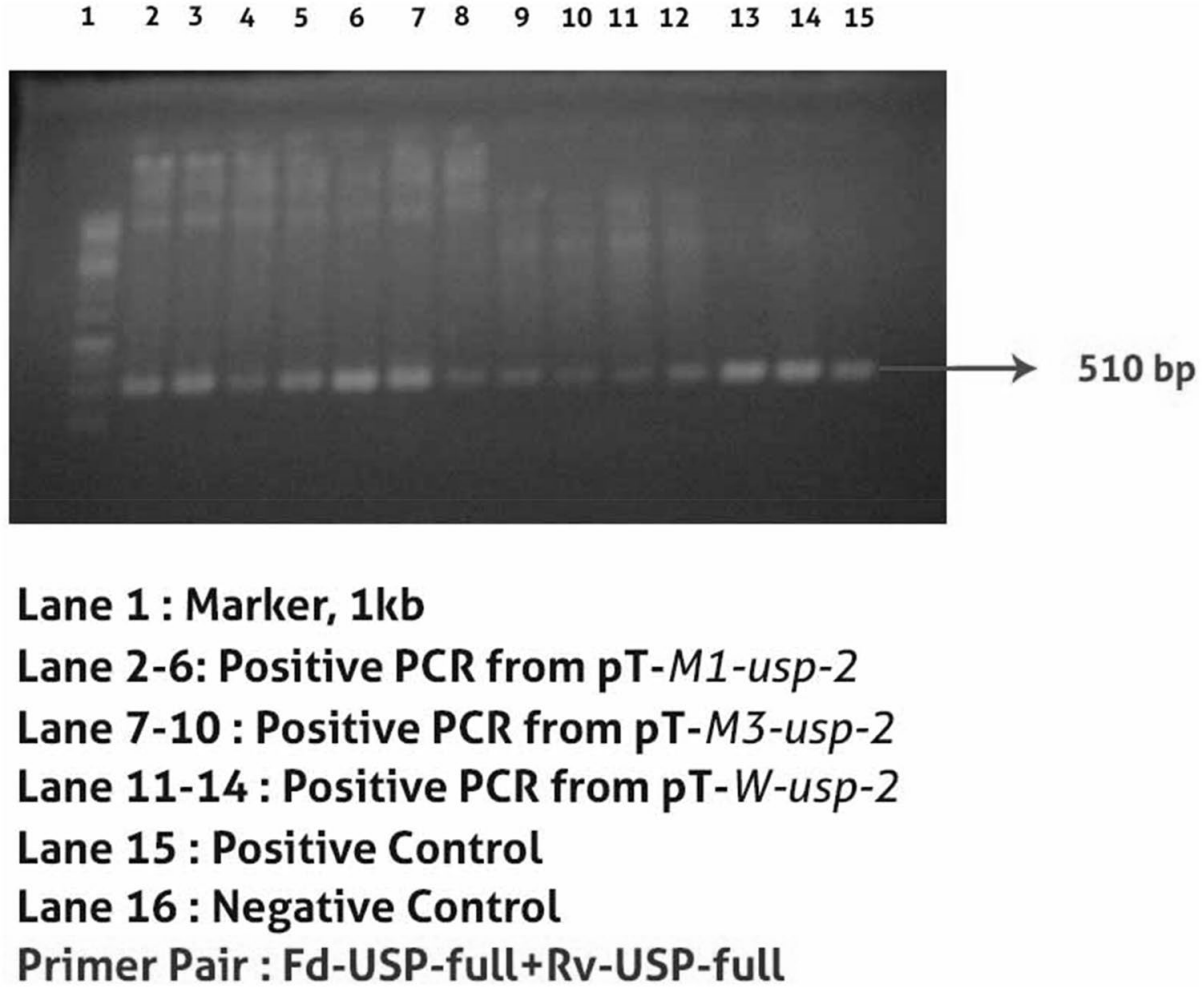
PCR amplification of *W-usp-2*, *M1-usp-2*&*M3-usp-2* in transgenic plants.

Total 16, 18, 19 transgenic plants of p*W-usp-2*, p*M1-usp-2* and p*M3-usp-2* respectively were confirmed PCR amplification (Fig. 9). The transgenic plants of wild type *GUSP-2* was named pT-*W-usp-2,* similarly, transgenic plants of p*M1-usp-2* and p*M3-usp-2* was labeled as pT-*M1-usp-2* and pT-*M3-usp-*2 respectively. All transgenic plants were found phenotypically healthy and their growth rate was normal.

### Molecular analysis of transgenic cotton plants

**Figure 10:**
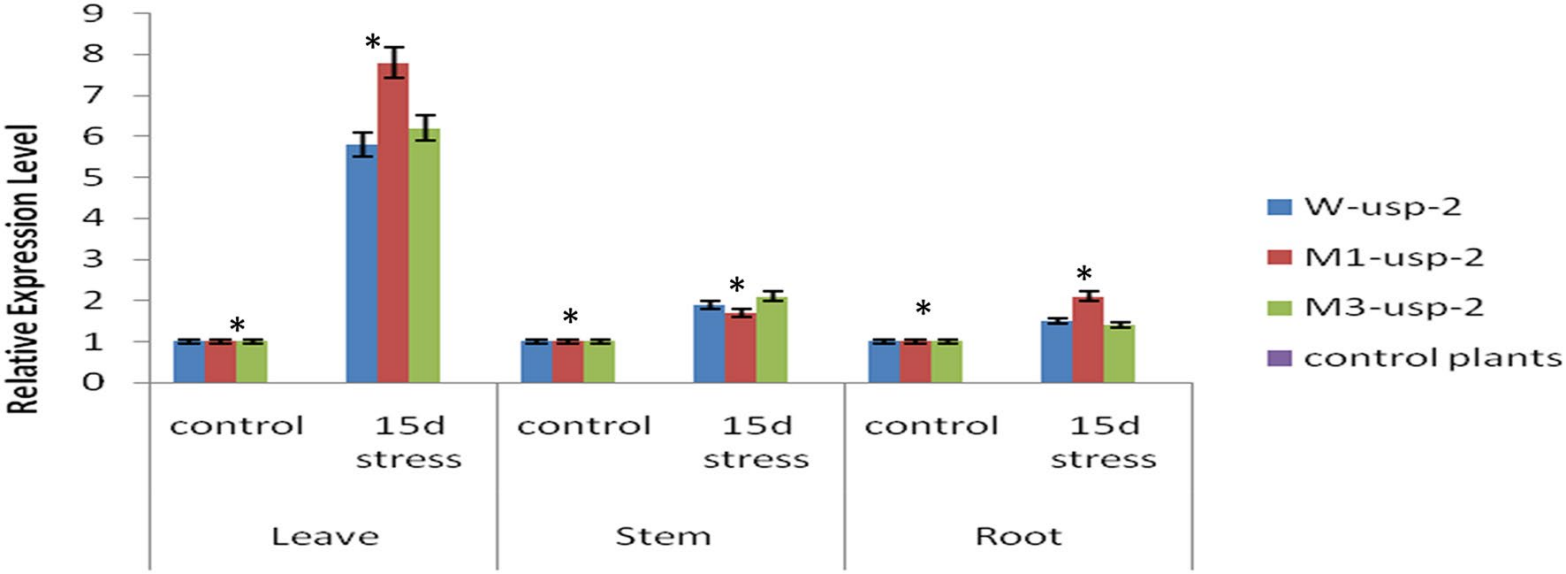
Spatial Expression of wild type and mutated *GUSP-2* genes in transgenic plants under drought stress conditions.

Expression analysis revealed that under drought stress condition both mutated and non-mutated *GUSP-2* genes were expressed higher in leaves as compared to root and stem. The leaves of drought stressed transgenic (pT-*M1-usp-2*) plants contained p*M1-usp-2* construct showed 7.8 folds expression of *M1-usp-2* as compared to (well-watered) control plants. The stem of same plants also showed 2.6 folds expression as compared to control and their roots also express *M1-usp-2* at 2.1 folds more than that of control plants. The expression of *M3-usp-2* in leaves of transgenic plants (6.2 folds) was almost similar to the expression of *W-usp-2* wild type *GUSP-2* (5.8 folds). However, roots of pT-*M3-usp-2* and pT-*W-usp-2* transgenic plants showed 1.4 to 1.5 folds more expression as compared with control. Similarly, stem of both transgenic plants expressed respective transformed genes at 1.7–1.9 folds more than that of controls. Both mutated and wild type *GUSP-2* genes were more expressed in leaves of transgenic plants but the expression of *M1-usp-2* was relatively higher in pT-*M1-usp-2* (Fig. 10).

### Quantification of protein with ELISA

**Figure 11:**
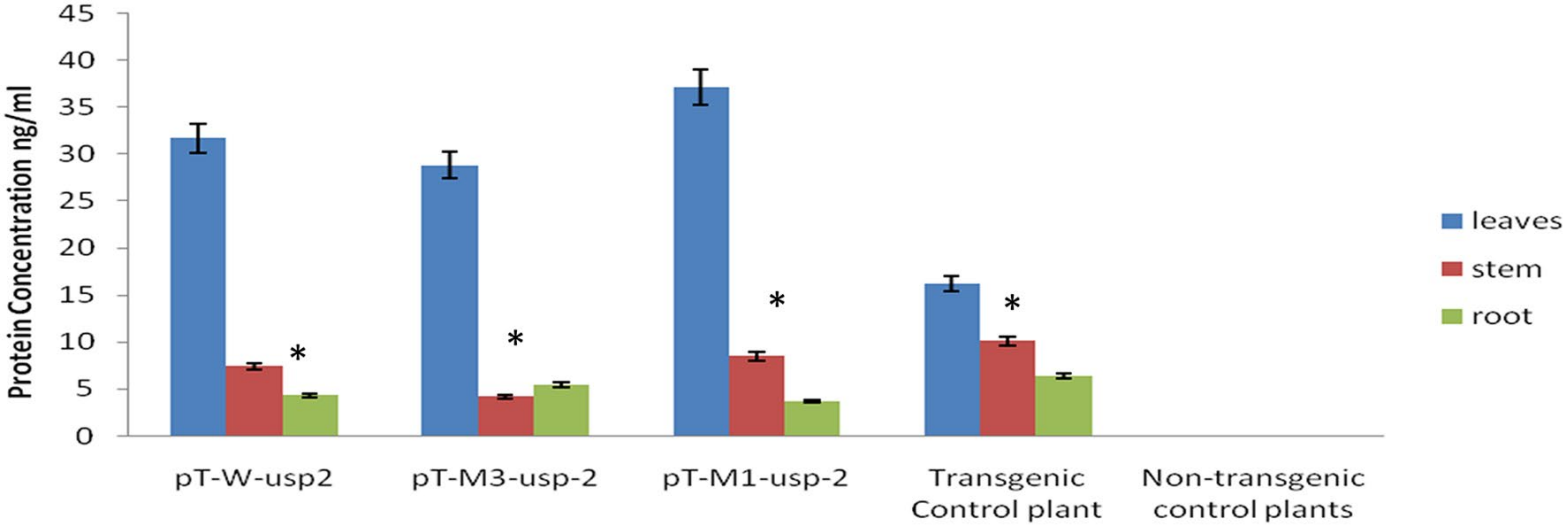
Concentration of wild type and mutated GUSP-2-proteins in different tissues of pT-*W-usp-2*, pT- *M1-usp-2*& pT-*M3-usp-2* transgenic cotton plants.

The protein concentration was found more in drought stressed leaves as compared with roots and stems of all transgenic plants. The concentration mutated protein (*M1-usp-2*) was slightly more than that of wild type (*W-usp-2*) and mutant-3 (*M3-usp-2*) protein. The protein in leaves of drought stressed transgenic cotton plants (pT-*W-usp-2,* pT-*M1-usp-2 &* pT-*M3-usp-2*) was found at 31.7, 37.1 and 28.8 ng/ml respectively. However, protein concentration in leaves of control plants was observed at 16.2 ng/ml. Both wild type and mutated *GUSP-2* proteins in roots and stems of transgenic plants was found closer to that of control plant (Fig. 11).

### Morphological analysis of transgenic plants

#### Plant height

**Figure 12:**
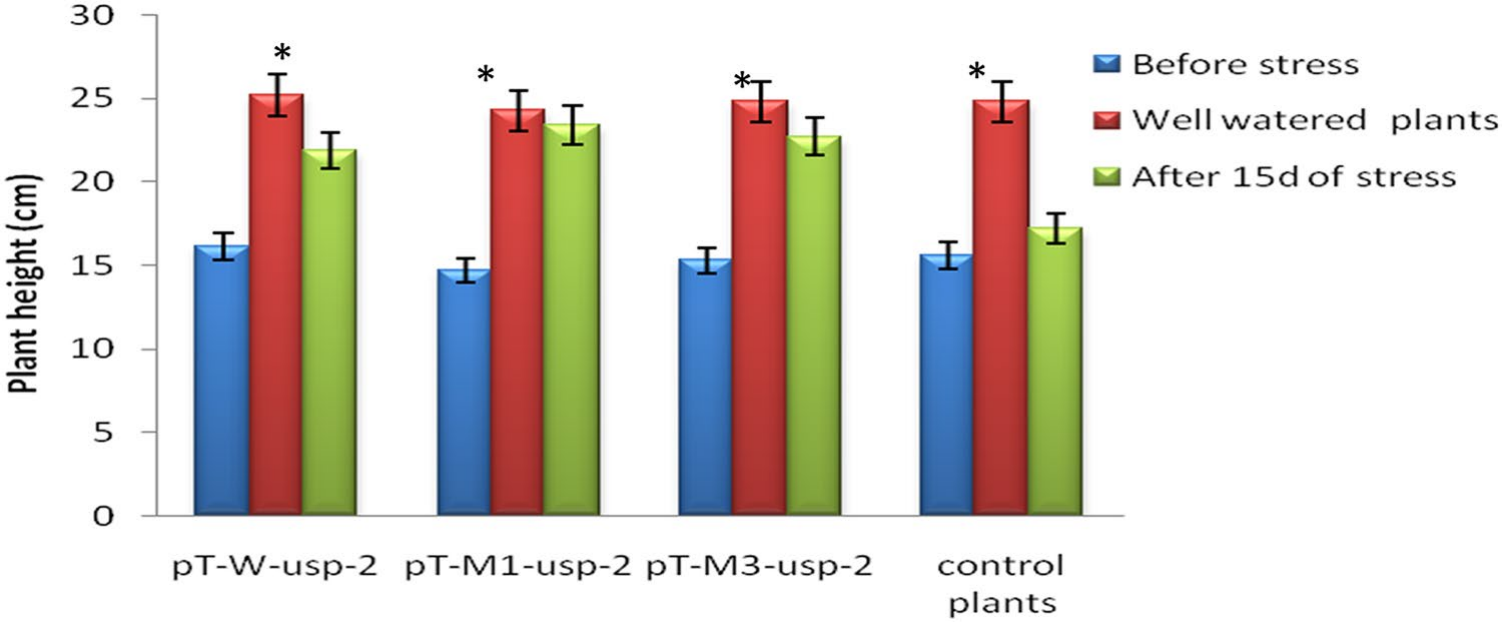
Comparison of Plant height (cm) of pT-*W-usp-2*, pT-*M1 -usp-2*, pT-*M3-usp-2* plants under control and drought stress conditions.

The initial plant height of control plants before the application of stress treatment was noted as 15.6 cm. Similarly, initial plant height of transgenic plants (pT-*W-usp-2*) transformed with p*W-usp-2* construct was 16.1 cm. The initial heights of transgenic plants (pT-*M1 -usp-2* & pT-*M3-usp-2*) transformed with p*M1-usp-2* & p*M3-usp-2* constructs was note down as 14.7 cm and 15.3 cm respectively. After 15d of drought stress the height of pT-*W-usp-2,* pT-*M -usp-2* and pT-*M3-usp-2* plants were observed as 21.9, 23.4 and 22.7 cm respectively. However, the height of non-transgenic *CIM-496* was increased as 17.2 cm after 15d of drought stress. Although, after 15d the final height of control transgenic and control non-transgenic plants were 25.2, 24.3, 24.8 and 24.8 cm for pT-*W-usp-2,* pT-*M1 -usp-2,* pT-*M3-usp-*2 and control plants respectively (Fig. 12).

#### Root length

**Figure 13:**
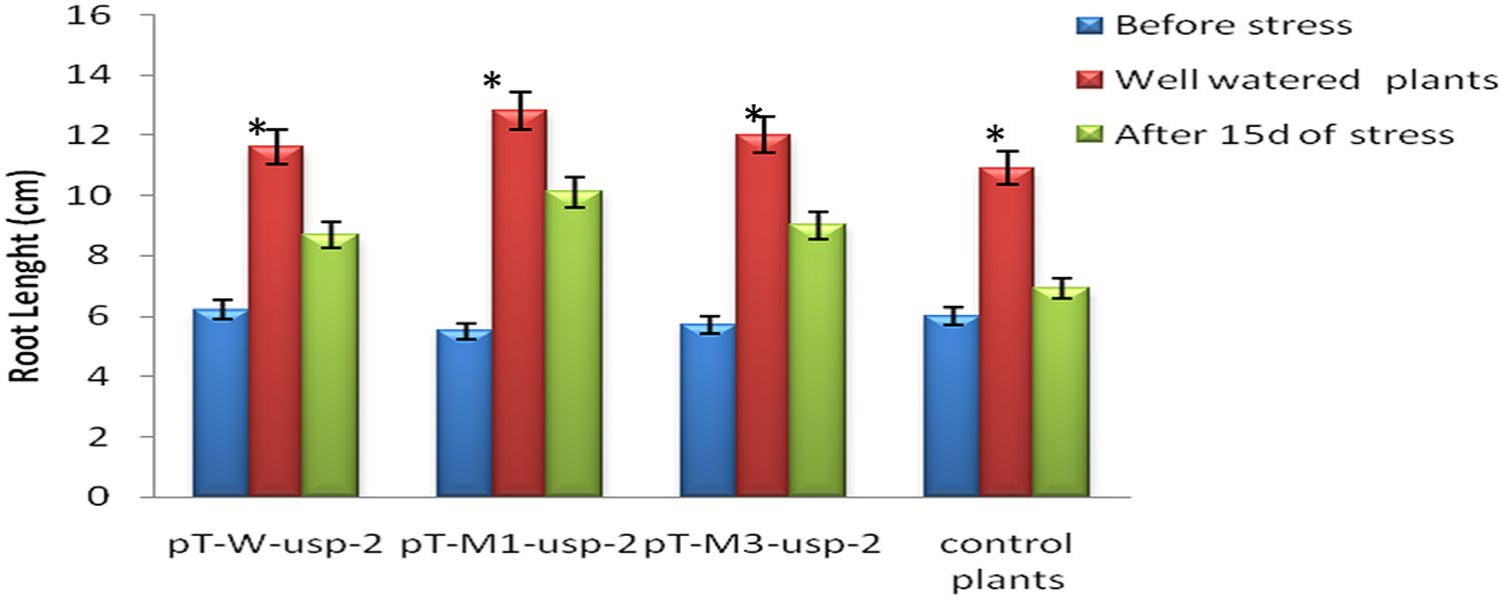
Comparison of Root Length (cm) of pT-*W-usp-2*, pT-*M1 -usp-2*, pT-*M3-usp-2* plants under control and drought stress conditions.

Root length of transgenic plants (pT-*W-usp-2,* pT-*M1 -usp-2* & pT-*M3-usp-2*) before stress treatment was 6.2, 5.5, 5.7 cm respectively. While, root length of non-transgenic control plants was noted as 6 cm. The transgenic control plants growing under control conditions, root length after 15d was 11.6, 12.8, 12 and 10.9 cm for pT-*W-usp-2,* pT-*M1 -usp-2,* pT-*M3-usp-*2 and control plants respectively (Fig. 13). After 15d, the root length of transgenic plants (pT-*W-usp-2,* pT-*M1 -usp-2* and pT-*M3-usp-2*) growing under drought stress was observed as 8.7, 10.1 and 9 cm respectively, while root length of non-transgenic plant after 15d of stress was 6.9 cm.

### Physiological analysis of transgenic plants

#### Relative water content

**Figure 14:**
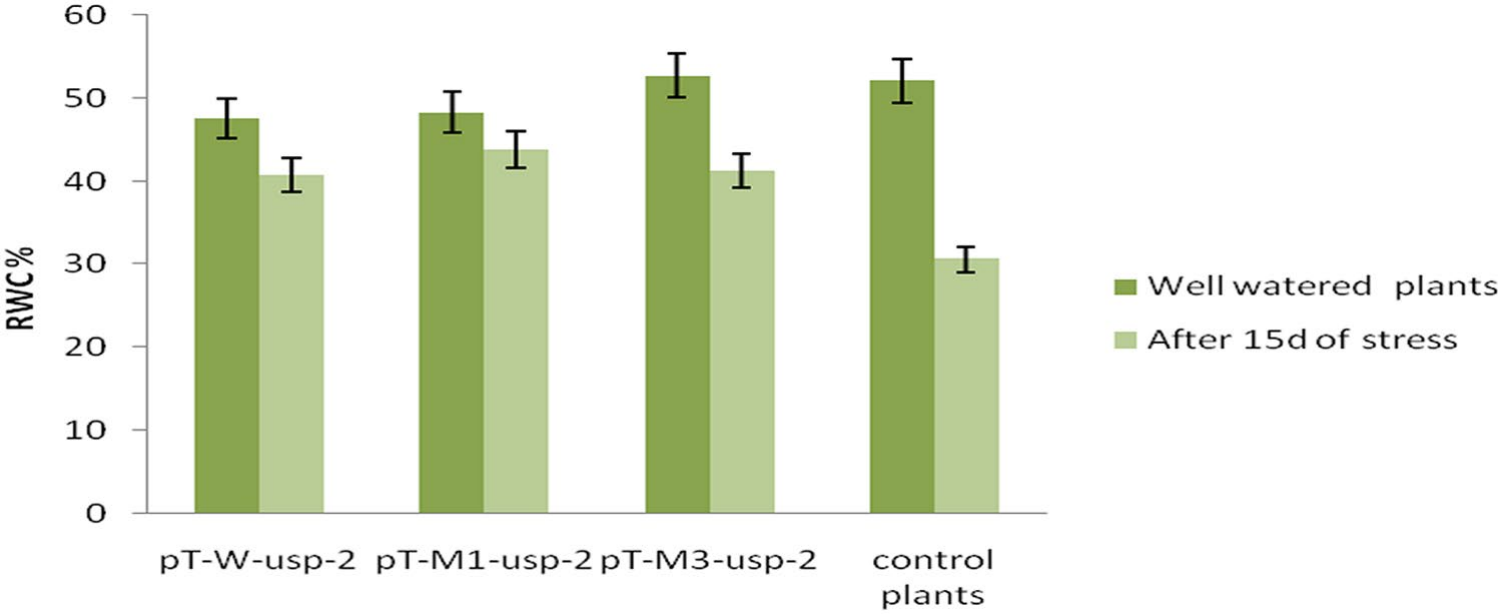
RWC of pT-*W-usp-2*, pT-*M1 -usp-2*, pT-*M3-usp-2* plants under control and drought stress conditions.

Relative water content (RWC) of transgenic cotton plants (pT-*W-usp-2,* pT-*M1 -usp-2* & pT-*M3-usp-2*) after 15d of stress treatment was 40.7, 43.8, 41.3% respectively while, RWC of control plants under same stress conditions was noted as 30.6 cm (Fig. 14). However, RWC of transgenic and non-transgenic control plants growing under control condition was 47.6, 48.3, 52.7 and 52.1% respectively for pT-*W-usp-2,* pT-*M1 -usp-2,* pT-*M3-usp-2* and non-transgenic control plants. RWC of transgenic plants (pT-*M3-usp-2,* pT-*W-usp-2*) contained mutated, p*M3-usp-2,* and non-mutated, p*W-usp-2,* constructs respectively was remained almost same (41.3% ≈ 40.7%) after 15d of drought stress but remained higher than that of control plants (30.6%). Transgenic plants, pT-*M1-usp-2,* performed well in terms of RWC under drought stress treatment (43.8%) as compared to 3rd mutated, non-mutated and control plants. Comparison between the leaf fresh weight with leaf turgor weight and dry weight revealed that the leaf relative water content was decreased under drought stress treatment more rapidly in non-transgenic plants then that of transgenic plants especially pT-*M1-usp-2.*

#### Photosynthetic activity

**Figure 15:**
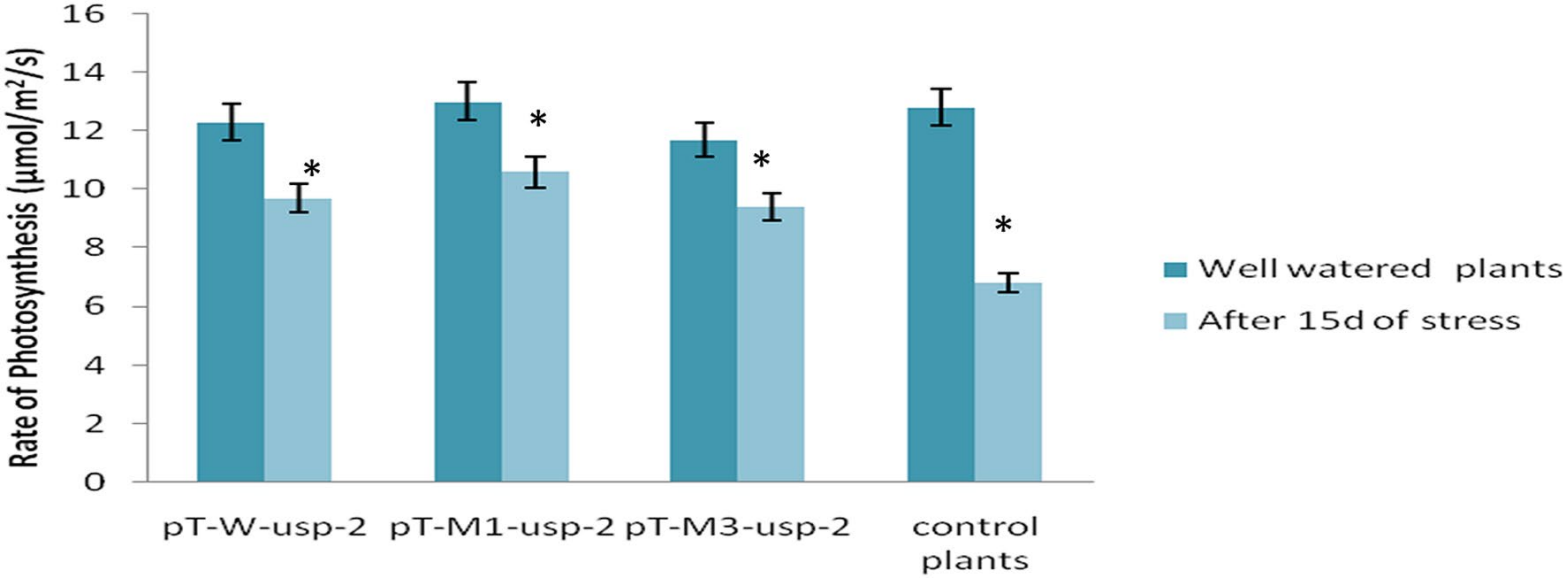
Photosynthetic rate of pT-*W-usp-2*, pT-*M1-usp-2*, pT-*M3-usp-2* plants under control and drought stress conditions.

We observed 10.6 µmol m^−2^ s^−1^ maximum photosynthesis rate for transgenic plants contained mutated (p*M1-usp-2*) *GUSP-2* gene under drought stress of 15d. However, photosynthetic activity of same transgenic plants was observed as 13 µmol m^−2^ s^−1^ under control conditions. Photosynthesis of regularly watered transgenic plants containing mutated construct (p*M3-usp-2*) and non-mutated construct (p*W-usp-2*) was noted as 11.7, 12.3 µmolm^−2^ s^−1^ respectively and under drought stress treatment their photosynthetic rate was decreased to 9.7, 9.4*µ*molm^-2^ s^−1^ but remain almost equal. The photosynthesis activity was decreased to 6.8 from 12.8 µmolm^−2^ s^−1^ under control to stressed conditions (Fig. 15). With the application of drought stress treatment, photosynthetic activity was observed to be decreased in non-transgenic control plants as compared to transgenic plants. However, among transgenic plants pT-*M1-usp-2* (10.6 µmolm^−2^ s^−1^) rated at higher level as compared to pT-*W-usp-2* and pT-*M3-usp-2* (9.4 µmolm^−2^ s^−1^≈9.7 µmolm^−2^ s^−1^) plants.

### Biochemical analysis of transgenic plants

#### Proline content

**Figure 16:**
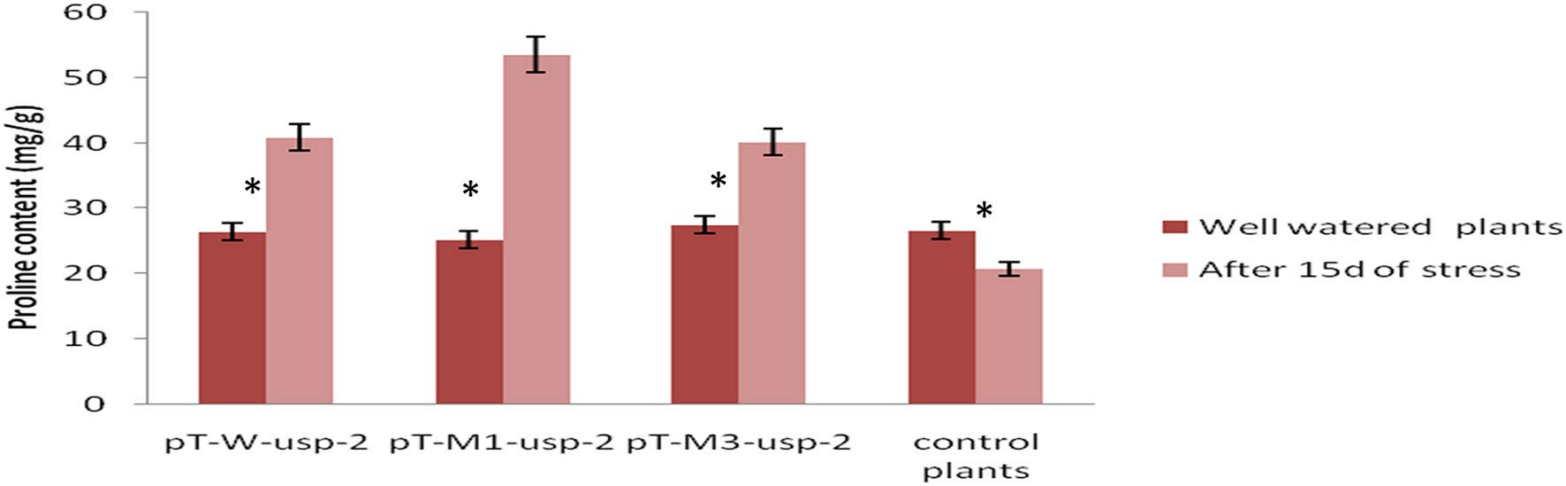
Comparison of Proline content from pT-*W-usp-2*, pT-*M1-usp-2*, pT-*M3-usp-2* plants under control and drought stress conditions.

The concentration of proline was found higher in transgenic plants, which were subjected to drought stress conditions as compared to the plants under control conditions. Minimum proline contents were observed as 25.2 mg/g under control condition for transgenic plant containing mutated construct (p*M1-usp-2*) and after 15d of stress the contents were noted as 43.6 mg/g for the same plant. Proline content of regularly watered transgenic plants containing mutated construct, p*M3-usp-2,* and non-mutated construct, p*W-usp-2,* were noted as 27.5, 26.4 mg/g respectively, however, it was increased up to 40.2, 40.8 mg/g after 15d of drought stress. For controls non-transgenic plants, it was 26.6 mg/g under regular watering conditions and it was decreased to 20.86 mg/g under drought stress treatment. With the application of drought stress treatment, proline content was increased in transgenic plants but decreased in non-transgenic plants (Fig. 16). When comparing among transgenic plants, proline content from leaves of pT-*M1-usp-2* transgenic plants containing p*M1-usp-2* mutated construct was highest (51.5 mg/g) as compared to pT-*M3-usp-2*plants (40.2 mg/g), pT-*W-usp-2* plants (40.86 mg/g). The proline contents from pT-*W-usp-2* plants pT-*M3-usp-2* plants were observed almost same (40.86 mg/g ≈ 40.26 mg/g).

#### Confocal microscopy for localization of *GUSP-2* with GFP

**Figure 17:**
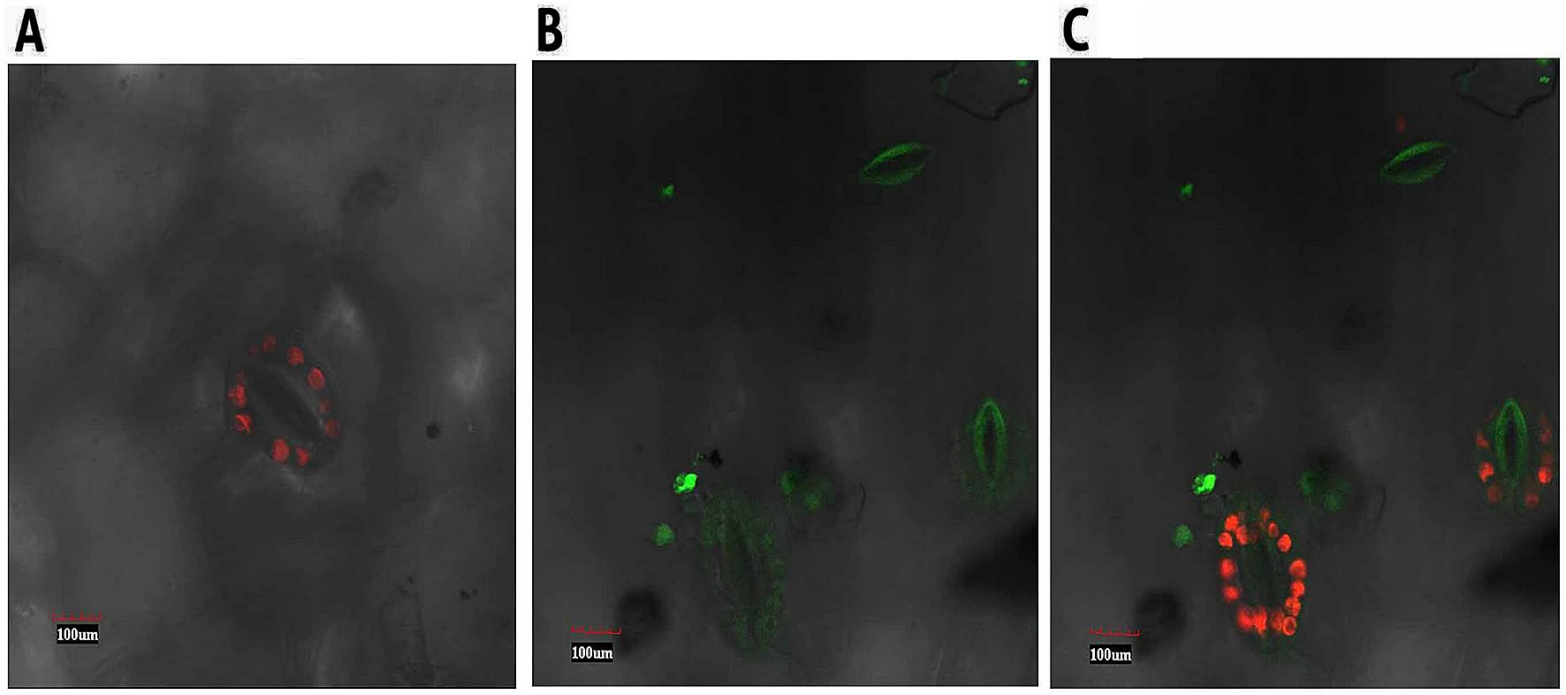
Confocal microscopy for localization of *GUSP-2* in leaves of transgenic plants, (**A**) control plant, (**B**) transgenic plant with GFP fluorescence (**C**) transgenic plant with GFP fluorescence and chloroplast fluorescence.

Wild type *GUSP-2* gene (*W-usp-2*) was observed localized with *GFP* fluorescence in the leaves of pT-*W-usp-2* transgenic plants by using confocal microscopy. With localization of green fluorescence, it was found that *W-usp-2,* non-mutated form of *GUSP-2,* was expressed in guard cells of stomata. Green fluorescence was not seen in the leaves of control plants, but red fluorescence of chloroplast was observed (Fig. 17A). Figure 17B, demonstrated the green fluorescent image of pT-*W-usp-2* leaves in guard cells of stomata, Fig. 17C is merged image of both showed green fluorescence of *GFP* and red fluorescence of chlorophyll. Close observation of confocal images of control and transgenic leaves also revealed that the guard cells were shrinked in which *GUSP-2* gene was expressed and stomata was seen closed. The guard cells with no *GUSP-2* expression were seen turgid and stomata were remained open. It means *GUSP-2* gene is expressing in guard cells of transgenic leaves and playing role in functioning of stomata under drought stress.

### Statistical analysis

Analysis of variance (ANOVA) for *E. coli* transformed with recombinant vectors containing *W-usp-2*, *M1-usp-2*, *M2-usp-2*, *M3-usp-2* genes showed significant differences under salt and osmotic stresses for mutant genes, vector control, wild type and cell control at *P* < 0.05 but no significant difference was observed under heat stress treatment. Comparative study among mutant genes showed no significant difference between *W-usp-2* and *M3-usp-2* *P* < 0.05 under all stress conditions (Supplementary Table S1). Similarly, analysis for morphological, physiological, biochemical and molecular data of transgenic plants (p*T-M1usp-2*, p*T-M3-usp-2,* p*T-W-usp-2*) transformed with mutated (p*M1-usp-2*, p*M2-usp-2*) and non-mutated (p*W-usp-2*) constructs and non-transgenic plants showed significant differences among mutated and non-mutated genotypes under drought stress treatment. However, comparison among mutant genes, revealed no significant difference between pT-*W-usp-2* and pT-*M3-usp-2* at *P* < 0.05 (Supplementary Table S2). Under control conditions, 25.2 cm plant (shoot) height was recorded for transgenic plants, while under drought stress conditions the mutant gene *M1-usp-2* (23.4 cm) showed higher expression in the form of long (higher) plant height. Significant difference was observed among mutant genes, stress and the interactions between gene × stress for morphological traits including shoot length (plant height) and root length (Supplementary Table S3). Major difference was found between p*T-M1-usp-2* (10.1, 24.4 cm, 0.43) and p*T-M3-usp-2* (9, 22.7 cm, 0.39) at *P* < 0.05 for root length and shot length but no significant difference was recorded between p*T-W-usp-2* (8.7, 21.9 cm, 0.38) and p*T-M3-usp-2* (9, 22.7 cm, 0.39).

Significant difference was also reported among mutant genes, stress and the interactions between gene × stress for physiological traits including relative water content, photosynthetic rate, (Supplementary Table S4). It was revealed from results that the relative water content (43.8%) was found higher under drought stress condition for p*T-M1-usp-2* as compared to other transgenic plants and non-transgenic (control) plants. Similarly, higher photosynthetic rate and lower transpiration rate and stomatal conductance were found for p*T-M1-usp-2* transgenic plants. It was observed significant difference among mutant genes, stress and the interactions between gene × stress for proline content *P* < 0.05 (Supplementary Table S5). The mean performance of genes under water stress condition indicated that mutant gene *M1-usp-2* initiated the formation of higher proline to improve drought stress tolerance and normal growth of transgenic plants. Similarly, significant difference was also recorded among mutant genes, stress and the interactions between gene × stress for ELISA analysis at *P* < 0.05 (Supplementary Table S6). It was found that M1-usp-2-protein was expressed at higher level (36.885 mg/g) in drought stressed leaves of p*T-M1-usp-2* as compared to the expression of 3rd mutant protein (M3-usp-2) in pT-M3-usp-2 (28.570 mg/g) and wild type protein (W-usp-2) in pT-W-usp-2 (31.630 mg/g).

## Discussions

We characterized cDNA clone of *GUSP-2* from *Gossypium arboreum* that encodes predicted 19.1 kDa protein*.* This protein has 81% homology with GUSP-1*,* another protein from cotton. GUSP-2 has two conserved domain, one at N-terminus and other at C-terminus and both are different from each other. This difference may partially explain why this protein has resistance against stress conditions. Interaction of GUSP-2-potein with adenosine monophosphate and the presence of glycosylation, phosphorylation and ATP-binding sites (predicted by *in-silico* analysis) suggested its involvement in signal transduction. Previously, it was observed that rice OsUSP1 belongs to subfamily of ATP-binding USP, and plant USPs might contain ATP binding domain dimmers^25–27^.

The functinal acitivity of GUSP-2-protein was enhanced by site directed mutations. Three different mutant of GUSP-2 proteins were produced by point mutated the wild type *GUSP-2* gene at three different positions^21,28^. The predicted model of GUSP-2 was used as reference to insert three mutations by using MOE tool. By replacing Lysine with proline in mutant-1 (M1-usp-2) its ATP binding capacity was enhanced by 2× and 2nd mutant protein (M2-usp-2) was abnormal with zero ATP-binding capacity, however, CMP capacity of 3rd mutant protein (M3-usp-2) was enhanced by replacing Lysine with Threonine residue. The interaction of 1st and 3rd GUSP-2 mutated proteins with 2gm3. A template was increased by increase in number of the hydrogen bonds, so it was predicted that functional activity of GUSP-2 protein will be enhanced. Previously, it was studied that activity of Mycobacterium-USP-protein was enhanced by enhancing its ATP-binding capacity and it became more virulent^29–32^.

The GUS has been replaced in pMV vector with 550 bp *SpUSP* gene from tomato which exhibited significant tolerance to heat, cold, drought and salt stress^33,34^. *G. arboreum* is known for its resistance against abiotic and biotic stresses, it has priceless gene pool to improve future cotton cultivar^8,32^. Similarly, two genotypes of cotton (*FDH-786* and *FDH-171)* studied by Hassan et al*.*^5^ under 800 mM NaCl stress and declared both are tolerant to salt stress and can be utilized for the improvement of traits in crops. In a comparative study, *CIM-496 G. hirsutum* was reported drought susceptible cultivar as compared to *FDH-786 G. arboreum*^4^. Several barley stress related genes (*USP*-gens) were reported to express under NaCl stress by Li et al*.*^35^. *SbUSP-*gene 783 bp from *Salicornia brachiata* was expressed in *E. coli* which conferred to osmotic and salt stress^36^. Previously, three salt responsive genes cloned from *S. brachiata* showed significant expression under abiotic stresses in host plant. Among them expression of *SbMT-2* was maximum under salt (500 mM), heat (45 °C) treatments; however, expression decreased under cold stress^37^. Similarly, expression of *SbpAPX* gene was dercreased under osmatic stress but highest expression was noted under (NaCl 500 mM) stress^38^. The expression of *SbGST* gene was increased under different NaCl treatments^37^, however, expression of both *SbGST* and *SbpAPX* genes was strongly induced under cold stress^36,39^. *SbUSP* from *S. brachiata* was cloned and expressed under abiotic stress conditions in *E. coli BL-21-DE,* highest level of expression (7.1 fold) was observed under salt (800 mM) stress^40^.

M1-usp-2 protein enhanced survival rate of *E. coli BL-21-∆* as compared to cells expressed W-usp-2 and M2-usp-2, M3-usp-2 proteins. Previously, it was concluded that *SbUSP* from *S. brachiata* showed 1.3-fold lower accumulation of Na ions compared to control bacterial cells thus imparted salt tolerance to *E. coli-BL-21-DE* under salt stress^38^. Maqbool et al*.*^10^ reported elevated expression of *GUSP-1* and *GUSP-2* genes in leaves of *G. arboreum* under drought stress. Several other putative stress responsive genes were isolated and charachterized in barely^35^. Similarly, *USp* gene cloned from *solanum pennellii* was expressed under salt, heat and osmatic stress treatments. It was observed that *USP* involved in stress adaptation mechanisms under various abiotic stress conditions at cellular level in plants and these genes belonged to ubiquitous gene family.

In spot assay growth of cells heat or might be mutant-1 and wild type *GUSP-2* gene exhibited slight tolerance against heat and cold stresses. However, *LEA* gene cloned from *Pogonatherum paniceum* differentially affected bacterial tolerance to cold and heat stress^41^. Similarly, bacteria expressing *Os*LEA5 enhanced tolerance against various abiotic stress conditions including heat^42^ and E. coli transformed with *MuNAC4* gene demonstrated tolerance in saline medium but grow poorly at 46 °C^43^. Relative fold expression of *M1-usp-2* gene under 8% PEG stress in bacterial cells was observed at uppermost level (5.7 fold) as compared to *M2-usp-2* (1.8 fold) and *W-usp-2* (4.00 fold) which is almost similar to the expression level of *M3-usp-2* gene. However, under heat stress all genes did not expressed significantly either because of cell death or genes don’t confer resistance against heat stress. Stress associated proteins (SAPs) are concerned with stress related response of plants^28^ and universal stress protein (*USP*) genes provide tolerance against prolgonged abiotic stress condtions and their relative expression level was observed to be increased.

The transcription rate of *M1-usp-2, M3-usp-2* &*W-usp-2* under various stresses viz. salt and osmatic was enhanced. *M2-usp-2* expression in host organisms was also noticable but this mutated protein stay behind to impart significant tolerance. It means mutatnt-2 protein (dephosphorylated, zero ATP-binding capacity) failed to initiate any metabolic process of phosphorylated containing compound (signal transduction). However, mutant-1 (*M1-usp-2*) protein was found more active (enhanced phosphorylation or max ATP-binding ability) under salt and osmatic stresses as compared to wild type stress protein. Point mutated *GUSP-1* gene from *G. arboreum* was cloned and expressed in *G. hirsutum,* was more acitve (having more ATP-binding sites) and its expression was maximun in different parts of plants (root, stem, and leaves) as compared to control *GUSP-1* gene under drought stress^5^.

When transgenic plants containing mutated and non-mutated *GUSP-2* genes were compared with each other, pT*M1-usp-2* was found more stress tolerant on the basis of their morphological characteristics. Reduction in stem length in Soya bean plants was reported by Specht et al*.*^44^. Similarly, 25% reduction in plant height was observed in water stressed citrus seedlings by Wu and Xia^45^. Significant reduction in the stem length of potato plant was also reported under drought stress. The height of other plant species like *Abelmoschus esculentus*^46^, *Vigna unguiculata*^47^ and *Petrosolinum crispum*^48^ were reported to be reduced under drought stress. Ferreira et al*.*^49^ reported that progressive decline in root to shoot length ratio is because of drought stress in plants especially in *G. hirsutum*. As drought stress directly affects leaves, so, measurement of relative water content (RWC) and photosynthetic rate were included in this study to measure the degree of drought stress tolerance in transgenic *CIM-496 G. hirsutum* plants. Ferreira et al*.*^49^ reported that progressive decline in RWC is because of drought stress in plants especially in *Gossypium hirsutum*. Assaad and Signer^50^ found positive relationship between RWC and leaf water content, however, when the stress is disappeared, RWC progressively recovered within 48 h. High temperature and drought stress alters the structure of membrane proteins which enhanced the permeability of membrane and increased the loss of electrolyte^51^. The increased solute leakage has been used as indirect measure of drought stress effects. It has been studied by Chen et al*.*^52^ in potato and tomato, in cotton by Maqbool et al.^53^, in winter wheat by Martin et al*.*^54^, in cotton by Ashraf et al.^55^, in sorghum by Marcum^56^, in cowpea by Ismail and Hall^57^ and in barley by Waheed and Shabir^58^.

Proline is considered as a compatible solute as well as osmo-protectant, it protects the plant tissues by producing stress responsive protein^59^. Kumar and Reddy^60^ revealed that, when water potential becomes the amount of osmolytes which are imperative for osmoregulation, allows additional water from environment. This helps in minimizing the immediate effect of drought stress. Similarly, Unyayar and Keles^61^, while studying the characteristics of *Helianthus annus* under drought condition observed a strong correlation between proline content and water deficiency. Proline also play role to stabilize membranes and cellular proteins in the presence of high level of osmoticum^62^. The results of current study are in accordance with the findings of Krasichkova et al.^63^ and Mohammadian and Moghaddam^64^ who reported that proline and sugar contents were increased under drought stress in drought tolerant plants. Elevated proline level in wheat plant reported under drought stress by Vereyken et al.^65^. Therefore, elevated concentration of USPs in cotton might help in maintaining the proline synthesis which play role to stabilize membranes and cellular proteins.

Real Time expression analysis revealed that wild type and mutated forms of GUSP-2 genes were expressed in all parts of plants, but maximum expression was found in leaves. Comparative analysis showed that expression of *M1-usp-2* was maximum (7.8 folds) in leaves as compared to *M3-usp-2* (6.2 folds) and *W-usp-2* (5.8 folds). Udawat et al*.*^37^ reported increase in the transcription of *SbUSP*-gene under abiotic stress treatments. In chimeric proteins, GFP can fuse at C or N-terminus enable the transcription of *GFP* under same regulatory sequence as for target gene^66–68^. The GFP was cloned in fusion with mutated and wild type *GUSP-2* in pCAMBIA-1301b by replacing GUS with GFP. Likewise, GFP has been used to describe the localization of RAP2. Zinc finger protein-1 and antiphagocytic protein-1 with *GFP* from rice to identify their cellular localization in leaves of tobacco and tomato^69^.The leaves of CIM-496 *G. hirsutum* transgenic plants demonstrated the elevated level of GUSP-2-GFP transgene which was localized into the guard cells of leaves through confocal microscopy. In this study, the expression of GUSP-2-protein is localized in guard cells, which is contrary to the findings of Loukehaich et al.^33^ as they reported cellular localization of *SpUSP* in nucleus of stomata and cell membrane of tomato leaves.

## Conclusion

*GUSP-2* gene from *Gossypium arboreum* exhibited salt and osmatic tolerance in *E. coli BL-21-uspABC* mutant strains. The mutant-1 (*M1-usp-2*) form of this gene was more active and encoded mutated universal stress protein of adenine nucleotide alpha hydrolases superfamily, which conferred more tolerance aginst salt and osmatic stresses, thus could be utilized as an important genetic resource for abiotic stress tolerance. The mutant-1 (M1-usp-2), mutant-3 (M3-usp-2) and wild type (W-usp-2) ATP bound proteins may function as a signaling molecule to activate stress inducible pathway or my be directly involved in salt and osmatic stress tolerance mechanisms. Stress dependent activation and deactivation are of immense importance for biotechnology applications. Further studies will focus on some more suitable side directed mutations to enhance its activity and transformation in plant to analyse its biochemical mode of action.

## Supplementary Information

Supplementary Information.
