# Supplementary material for: MutantGossypium universal stress protein-2 (GUSP-2) gene confers resistance to various abiotic stresses in E. coli BL-21 and CIM-496-Gossypium hirsutum
Source: Sci Rep. 2021 Oct 14;11:20466. doi: 10.1038/s41598-021-99900-x (PMC8516947; doi:10.1038/s41598-021-99900-x)
Supplement: Supplementary file 1 — Supplementary Information. [file 41598_2021_99900_MOESM1_ESM.docx]

**Mutant *Gossypium Universal Stress Protein-2* (*GUSP-2*) gene confers resistance to various abiotic stresses in *E. coli*** *BL-*21 **and *CIM-496-Gossypium hirsutum***

**Muhammad Nadeem Hafeez**^1, 2, 3, 4^***, Mohsin Ahmad Khan**^1^**, Bilal Sarwar**^1^**, Sameera Hassan**^1^, **Qurban Ali**^1,5^*** Tayyab Husnain^1^, and Bushra Rashid**^1^

^1^Centre of Excellence in Molecular Biology, University of the Punjab Lahore, Pakistan

^2^Drug Research Program, Division of Pharmaceutical Chemistry and Technology, Faculty of Pharmacy, University of Helsinki, Helsinki, Finland

^3^School of PhD program in Cellular and Molecular Biotechnology, University of Teramo, Italy

^4^Department of Pharmacy, University of Chieti – Pescara “G. d’Annunzio”, Chieti, Italy ^5^Institute of Molecular Biology and Biotechnology, University of Lahore, Lahore, Pakistan

Corresponding author: [chnadeemhfz@gmail.com](mailto:chnadeemhfz@gmail.com), [saim1692@gmail.com](mailto:saim1692@gmail.com)

**Supplementary Materials**

**Tables S1: Analysis of variance and LSD for *E. coli* under Salt stress**

**Analysis of Variance Table under two factor factorial design for Cell control**

**Source DF SS MS F P**

Gene 2 0.00002 0.00001 0.01 0.0001

Time 5 1.81512 0.36302 231.52 0.0000

Gene*Time 10 0.00864 0.00086 0.55 0.0000

Error 18 0.02822 0.00157

Total 35 1.85201

Grand Mean 0.5574 CV 7.10

**Analysis of Variance Table under two factor factorial design for mutant genes**

**Source DF SS MS F P**

Gene 2 1.38249 0.69124 440.84 0.0000

Time 5 5.92126 1.18425 755.26 0.0000

Gene*Time 10 0.96724 0.09672 61.69 0.0000

Error 18 0.02822 0.00157

Total 35 8.29921

Grand Mean 0.7586 CV 5.22

**Analysis of Variance Table under two factor factorial design for Vector control**

**Source DF SS MS F P**

Gene 2 0.00009 0.00004 0.03 0.0001

Time 5 2.34476 0.46895 299.08 0.0000

Gene*Time 10 0.00364 0.00036 0.23 0.0006

Error 18 0.02822 0.00157

Total 35 2.37671

Grand Mean 0.5124 CV 7.73

**Analysis of Variance Table under two factor factorial design for *W-usp-2***

**Source DF SS MS F P**

Gene 2 0.02276 0.01138 7.26 0.0049

Time 5 8.65126 1.73025 1103.48 0.0000

gene*Time 10 0.10684 0.01068 6.81 0.0002

Error 18 0.02822 0.00157

Total 35 8.80908

Grand Mean 0.8819 CV 4.49

**LSD All-Pairwise Comparisons Test of Cell control**

**Gene Mean Homogeneous Groups**

1 0.5780 A

2 0.5380 B

3 0.5263 C

Alpha 0.05 Standard Error for Comparison 0.0162

**LSD All-Pairwise Comparisons Test of Cell control**

**Time Mean Homogeneous Groups**

12 0.8713 A

10 0.7747 B

8 0.6113 C

6 0.4847 D

4 0.3947 E

2 0.2080 F

Alpha 0.05 Standard Error for Comparison 0.0202

**LSD All-Pairwise Comparisons Test of mutant genes**

Gene Mean Homogeneous Groups

1 0.9397 A

3 0.8497 B

2 0.4863 C

Alpha 0.05 Standard Error for Comparison 0.0162

**LSD All-Pairwise Comparisons Test of mutant genes**

**Time Mean Homogeneous Groups**

12 1.4180 A

10 1.1580 A

8 0.7247 B

6 0.5613 BC

4 0.4147 BC

2 0.2747 C

Alpha 0.05 Standard Error for Comparison 0.1625

**LSD All-Pairwise Comparisons Test of Vector control**

**Gene Mean Homogeneous Groups**

1 0.5447 A

3 0.5013 B

2 0.4713 C

Alpha 0.05 Standard Error for Comparison 0.0162

**LSD All-Pairwise Comparisons Test of Vector control**

**Time Mean Homogeneous Groups**

12 0.9480 A

10 0.7013 B

8 0.5213 C

6 0.4047 D

4 0.3380 E

2 0.1613 F

Alpha 0.05 Standard Error for Comparison 0.0188

**LSD All-Pairwise Comparisons Test of *W-usp-2***

**Gene Mean Homogeneous Groups**

1 0.8997 A

2 0.8997 A

3 0.8463 B

Alpha 0.05 Standard Error for Comparison 0.0162

**LSD All-Pairwise Comparisons Test of *W-usp-2***

**Time Mean Homogeneous Groups**

12 1.6647 A

10 1.3447 B

8 0.9113 C

6 0.6447 D

4 0.4613 E

2 0.2647 F

Alpha 0.05 Standard Error for Comparison 0.0419

**Tables S2: Analysis of variance and LSD for *P. pastoris* under Salt stress**

**Completely Randomized AOV under two factor factorial design for Cell**

**Source DF SS MS F P**

replication 1 0.00360 0.00360

Gene 2 8.889E-05 4.444E-05 1.5E+29 0.0000

Time 5 1.52916 0.30583 1.0E+33 0.0000

Gene*Time 10 7.111E-04 7.111E-05 2.5E+29 0.0000

Error 17 4.834E-33 2.843E-34

Total 35 1.53356

Grand Mean 0.3989 CV 3.04

Standard Error of a Mean 4.944E-03

**Completely Randomized AOV under two factor factorial design for *M2-usp-2***

**Source DF SS MS F P**

replication 1 0.00360 0.00360

Gene 2 2.51349 1.25674 6.7E+32 0.0000

Time 5 4.35899 0.87180 4.6E+32 0.0000

Gene*Time 10 1.22998 0.12300 6.5E+31 0.0000

Error 17 3.210E-32 1.888E-33

Total 35 8.10606

Grand Mean 0.5939 CV 9.51

Standard Error of a Mean 0.1443

**Completely Randomized AOV under two factor factorial design for Vector control**

**Source DF SS MS F P**

replication 1 0.00360 0.00360

Gene 2 0.00180 9.000E-04 1.6E+30 0.0000

Time 5 2.73067 0.54613 9.6E+32 0.0000

Gene*Time 10 0.00393 3.933E-04 6.9E+29 0.0000

Error 17 9.658E-33 5.681E-34

Total 35 2.74000

Grand Mean 0.4300 CV 4.10

Standard Error of a Mean 7.201E-03

**Completely Randomized AOV under two factor factorial design for *W-usp-2***

**Source DF SS MS F P**

replication 1 0.00360 0.00360

Gene 2 2.000E-04 1.000E-04 4.6E+28 0.0000

Time 5 7.42903 1.48581 6.9E+32 0.0000

Gene*Time 10 0.00207 2.067E-04 9.5E+28 0.0000

Error 17 3.687E-32 2.169E-33

Total 35 7.43490

Grand Mean 0.8350 CV 1.67

Standard Error of a Mean 5.709E-03

**LSD All-Pairwise Comparisons Test of Cell control**

**Gene Mean Homogeneous Groups**

1 0.6210 A

2 0.5210 B

3 0.5103 C

Alpha 0.05 Standard Error for Comparison 0.0012

**LSD All-Pairwise Comparisons Test of Cell by Time**

**Time Mean Homogeneous Groups**

16 0.6467 A

18 0.6167 B

12 0.4533 C

10 0.3767 D

8 0.2467 E

4 0.0533 F

Alpha 0.05 Standard Error for Comparison 6.992E-03

**LSD All-Pairwise Comparisons Test of mutant genes**

**Gene Mean Homogeneous Groups**

1 0.7397 A

3 0.6497 B

2 0.4363 C

Alpha 0.05 Standard Error for Comparison 0.0621

**LSD All-Pairwise Comparisons Test of mutant genes by Time**

**Time Mean Homogeneous Groups**

18 1.0733 A

16 0.9800 AB

12 0.6467 BC

10 0.4633 CD

8 0.2933 CD

4 0.1067 D

Alpha 0.05 Standard Error for Comparison 0.2040

**LSD All-Pairwise Comparisons Test of Vector control**

**Gene Mean Homogeneous Groups**

1 0.6147 A

3 0.5413 B

2 0.4113 C

Alpha 0.05 Standard Error for Comparison 0.0102

**LSD All-Pairwise Comparisons Test of Vector control by Time**

**Time Mean Homogeneous Groups**

18 0.7767 A

16 0.7533 B

12 0.5300 C

10 0.2800 D

8 0.1733 E

4 0.0667 F

Alpha 0.05 Standard Error for Comparison 0.0102

**LSD All-Pairwise Comparisons Test of *W-usp-2***

**Gene Mean Homogeneous Groups**

1 0.7197 A

2 0.6097 B

3 0.5413 C

Alpha 0.05 Standard Error for Comparison 0.0302

**LSD All-Pairwise Comparisons Test of *W-usp-2* by Time**

**Time Mean Homogeneous Groups**

18 1.3900 A

16 1.2167 B

12 1.1367 C

10 0.7167 D

8 0.4367 E

4 0.1133 F

Alpha 0.05 Standard Error for Comparison 8.074E-03

**Tables S3: Analysis of variance and LSD for *E. coli* under PEG stress**

**Completely Randomized AOVA under two factor factorial design for Cell control**

**Source DF SS MS F P**

Gene 2 0.00056 0.00028 0.15 0.0001

Time 5 2.56126 0.51225 284.58 0.0000

Gene*Time 10 0.00358 0.00036 0.20 0.0007

Error 18 0.03240 0.00180

Total 35 2.59779

Grand Mean 0.5594 CV 7.58

**Analysis of Variance Table under two factor factorial design for mutant genes**

**Source DF SS MS F P**

Gene 2 1.34516 0.67258 373.65 0.0000

Time 5 6.93796 1.38759 770.88 0.0000

Gene*Time 10 1.00004 0.10000 55.56 0.0000

Error 18 0.03240 0.00180

Total 35 9.31556

Grand Mean 0.8011 CV 5.30

**Analysis of Variance Table under two factor factorial design for Vector control**

**Source DF SS MS F P**

Gene 2 0.00036 0.00018 0.10 0.0004

Time 5 2.12099 0.42420 235.67 0.0000

Gene*Time 10 0.00684 0.00068 0.38 0.0001

Error 18 0.03240 0.00180

Total 35 2.16059

Grand Mean 0.6106 CV 6.95

**Analysis of Variance Table under two factor factorial design for *W-usp-2***

**Source DF SS MS F P**

Gene 2 0.00500 0.00250 1.39 0.0008

Time 5 8.71573 1.74315 968.41 0.0000

Gene*Time 10 0.00127 0.00013 0.07 0.0001

Error 18 0.03240 0.00180

Total 35 8.75440

Grand Mean 0.8600 CV 4.93

**LSD All-Pairwise Comparisons Test of Cell control**

**Gene Mean Homogeneous Groups**

1 0.5850 A

3 0.5267 B

2 0.5067 C

Alpha 0.05 Standard Error for Comparison 0.0173

**LSD All-Pairwise Comparisons Test of Cell control**

**Time Mean Homogeneous Groups**

12 1.0433 A

10 0.7133 B

8 0.5700 C

6 0.4500 D

4 0.3667 E

2 0.2133 F

Alpha 0.05 Standard Error for Comparison 0.0201

**LSD All-Pairwise Comparisons Test of mutant genes**

**Gene Mean Homogeneous Groups**

1 0.9933 A

3 0.8733 B

2 0.5367 C

Alpha 0.05 Standard Error for Comparison 0.0173

**LSD All-Pairwise Comparisons Test of mutant genes**

**Time Mean Homogeneous Groups**

12 1.5567 A

10 1.1767 B

8 0.7767 C

6 0.5633 CD

4 0.4367 D

2 0.2967 D

Alpha 0.05 Standard Error for Comparison 0.1625

**LSD All-Pairwise Comparisons Test of Vector control**

**Gene Mean Homogeneous Groups**

1 0.6150 A

3 0.5983 B

2 0.5783 C

Alpha 0.05 Standard Error for Comparison 0.0173

**LSD All-Pairwise Comparisons Test of Vector control**

**Time Mean Homogeneous Groups**

12 0.9633 A

10 0.8100 B

8 0.7000 C

6 0.5467 D

4 0.3933 E

2 0.2500 F

Alpha 0.05 Standard Error for Comparison 0.0210

**LSD All-Pairwise Comparisons Test of *W-usp-2***

**Gene Mean Homogeneous Groups**

1 0.8767 A

3 0.8417 B

2 0.8017 C

Alpha 0.05 Standard Error for Comparison 0.0173

**LSD All-Pairwise Comparisons Test of *W-usp-2***

**Time Mean Homogeneous Groups**

12 1.6833 A

10 1.2800 B

8 0.8767 C

6 0.6333 D

4 0.4233 E

2 0.2633 F

Alpha 0.05 Standard Error for Comparison 0.0207

**Tables S4: Analysis of variance and LSD for *P. pastoris* under PEG stress**

**Analysis of Variance Table under two factor factorial design for Cell**

**Source DF SS MS F P**

replication 1 0.00034 0.00034

Gene 2 0.00005 0.00003 0.01 0.9932

Time 5 1.90066 0.38013 103.37 0.0000

Gene*Time 10 0.00112 0.00011 0.03 1.0000

Error 17 0.06251 0.00368

Total 35 1.96468

Grand Mean 0.4358 CV 13.91

Standard Error of a Mean 0.0189

**Analysis of Variance Table under two factor factorial design for Vector**

**Source DF SS MS F P**

replication 1 0.01138 0.01138

Gene 2 0.00056 0.00028 0.07 0.9317

Time 5 3.46992 0.69398 177.35 0.0000

Gene*Time 10 0.00144 0.00014 0.04 1.0000

Error 17 0.06652 0.00391

Total 35 3.54982

Grand Mean 0.4878 CV 12.82

Standard Error of a Mean 0.0211

**Analysis of Variance Table under two factor factorial design for *W-usp-2***

**Source DF SS MS F P**

replication 1 0.06334 0.06334

Gene 2 0.00014 0.00007 0.00 0.9987

Time 5 8.98378 1.79676 34.45 0.0000

Gene*Time 10 0.00149 0.00015 0.00 1.0000

Error 17 0.88661 0.05215

Total 35 9.93536

Grand Mean 0.9169 CV 9.42

Standard Error of a Mean 0.0727

**Analysis of Variance Table under two factor factorial design for mutant genes**

**Source DF SS MS F P**

replication 1 0.00203 0.00203

Gene 2 2.46136 1.23068 490.83 0.0000

Time 5 4.79611 0.95922 382.56 0.0000

Gene*Time 10 1.09291 0.10929 43.59 0.0000

Error 17 0.04263 0.00251

Total 35 8.39503

Grand Mean 0.6264 CV 7.99

Standard Error of a Mean 0.1414

**LSD All-Pairwise Comparisons Test of Cell control**

**Gene Mean Homogeneous Groups**

1 0.6810 A

3 0.5723 B

2 0.5134 C

Alpha 0.05 Standard Error for Comparison 0.0143

**LSD All-Pairwise Comparisons Test of Cell by Time**

**Time Mean Homogeneous Groups**

18 0.7317 A

16 0.6600 B

12 0.5183 C

10 0.3950 D

8 0.2350 E

4 0.0750 F

Alpha 0.05 Standard Error for Comparison 0.0267

**LSD All-Pairwise Comparisons Test of mutant genes**

**Gene Mean Homogeneous Groups**

1 0.8923 A

3 0.8334 B

2 0.5632 C

Alpha 0.05 Standard Error for Comparison 0.0131

**LSD All-Pairwise Comparisons Test of mutant genes by Time**

**Time Mean Homogeneous Groups**

18 1.1317 A

16 1.0083 A

12 0.7233 AB

10 0.4883 BC

8 0.2800 C

4 0.1267 C

Alpha 0.05 Standard Error for Comparison 0.2000

**LSD All-Pairwise Comparisons Test of Vector control**

**Gene Mean Homogeneous Groups**

1 0.7120 A

3 0.5334 B

2 0.5125 C

Alpha 0.05 Standard Error for Comparison 0.0323

**LSD All-Pairwise Comparisons Test of Vector by Time**

**Time Mean Homogeneous Groups**

18 0.8900 A

16 0.8133 B

12 0.6417 C

10 0.3200 D

8 0.1750 E

4 0.0867 F

Alpha 0.05 Standard Error for Comparison 0.0298

**LSD All-Pairwise Comparisons Test of *W-usp-2***

**Gene Mean Homogeneous Groups**

1 0.7733 A

3 0.6422 B

2 0.6234 C

Alpha 0.05 Standard Error for Comparison 0.0213

**LSD All-Pairwise Comparisons Test of W by Time**

**Time Mean Homogeneous Groups**

12 1.5600 A

18 1.3750 AB

16 1.1800 B

10 0.7350 C

8 0.5167 D

4 0.1350 E

Alpha 0.05 Standard Error for Comparison 0.1028

**Tables S5: Analysis of variance and LSD for *E. coli* under heat stress**

**Analysis of Variance Table under two factor factorial design for Cell control**

**Source DF SS MS F P**

Gene 2 0.01562 0.00781 17.36 0.0001

Time 5 0.84486 0.16897 375.49 0.0000

Gene*Time 10 0.05971 0.00597 13.27 0.0000

Error 18 0.00810 0.00045

Total 35 0.92829

Grand Mean 0.4244 CV 5.00

**Analysis of Variance Table under two factor factorial design for mutant genes**

**Source DF SS MS F P**

Gene 2 0.09469 0.04734 105.21 0.0000

Time 5 0.66249 0.13250 294.44 0.0000

Gene*Time 10 0.03744 0.00374 8.32 0.0001

Error 18 0.00810 0.00045

Total 35 0.80272

Grand Mean 0.4472 CV 4.74

**Analysis of Variance Table under two factor factorial design for Vector control**

**Source DF SS MS F P**

Gene 2 0.00549 0.00274 6.10 0.0095

Time 5 0.65059 0.13012 289.15 0.0000

Gene*Time 10 0.04184 0.00418 9.30 0.0000

Error 18 0.00810 0.00045

Total 35 0.70602

Grand Mean 0.4122 CV 5.15

**Analysis of Variance Table under two factor factorial design for *W-usp-2***

**Source DF SS MS F P**

Gene 2 0.00222 0.00111 2.47 0.0020

Time 5 0.73769 0.14754 327.86 0.0000

Gene*Time 10 0.01324 0.00132 2.94 0.0003

Error 18 0.00810 0.00045

Total 35 0.76126

Grand Mean 0.4461 CV 4.76

**LSD All-Pairwise Comparisons Test of Cell control**

**Gene Mean Homogeneous Groups**

1 0.4533 A

2 0.4150 B

3 0.3850 C

Alpha 0.05 Standard Error for Comparison 8.660E-03

**LSD All-Pairwise Comparisons Test of Cell control**

**Time Mean Homogeneous Groups**

10 0.6050 A

12 0.5983 A

8 0.4650 B

6 0.3783 C

4 0.3317 C

2 0.1683 D

Alpha 0.05 Standard Error for Comparison 0.0304

**LSD All-Pairwise Comparisons Test of mutant genes**

**Gene Mean Homogeneous Groups**

1 0.5133 A

3 0.4400 B

2 0.3883 C

Alpha 0.05 Standard Error for Comparison 8.660E-03

**LSD All-Pairwise Comparisons Test of mutant genes**

**Time Mean Homogeneous Groups**

12 0.5950 A

10 0.5750 A

8 0.5217 A

6 0.4383 B

4 0.3417 C

2 0.2117 D

Alpha 0.05 Standard Error for Comparison 0.0395

**LSD All-Pairwise Comparisons Test of Vector control**

**Gene Mean Homogeneous Groups**

1 0.4233 A

3 0.4183 A

2 0.3950 B

Alpha 0.05 Standard Error for Comparison 8.660E-03

**LSD All-Pairwise Comparisons Test of Vector control**

**Time Mean Homogeneous Groups**

10 0.5617 A

12 0.5017 B

8 0.4917 B

6 0.4250 C

4 0.3383 D

2 0.1550 E

Alpha 0.05 Standard Error for Comparison 0.0248

**LSD All-Pairwise Comparisons Test of *W-usp-2***

**Gene Mean Homogeneous Groups**

1 0.4817 A

3 0.4517 B

2 0.4050 C

Alpha 0.05 Standard Error for Comparison 8.660E-03

**LSD All-Pairwise Comparisons Test of *W-usp-2***

**Time Mean Homogeneous Groups**

10 0.5883 A

12 0.5750 AB

8 0.5450 B

6 0.4250 C

4 0.3583 D

2 0.1850 E

Alpha 0.05 Standard Error for Comparison 0.0162

**Tables S6: Analysis of variance and LSD for *P. pastoris* under heat stress**

**Completely Randomized AOV under two factor factorial design for Cell**

**Source DF SS MS F P**

replication 1 0.00360 0.00360

Gene 2 2.000E-04 1.000E-04 3.6E+29 0.0000

Time 5 0.56840 0.11368 4.1E+32 0.0000

Gene*Time 10 0.00100 1.000E-04 3.6E+29 0.0000

Error 17 4.664E-33 2.744E-34

Total 35 0.57320

Grand Mean 0.3033 CV 4.17

Standard Error of a Mean 5.164E-03

**Completely Randomized AOV under two factor factorial design for Vector Control**

**Source DF SS MS F P**

replication 1 0.00360 0.00360

Gene 2 8.000E-04 4.000E-04 1.1E+30 0.0000

Time 5 0.91383 0.18277 5.2E+32 0.0000

Gene*Time 10 0.00187 1.867E-04 5.4E+29 0.0000

Error 17 5.931E-33 3.489E-34

Total 35 0.92010

Grand Mean 0.3350 CV 4.31

Standard Error of a Mean 5.900E-03

**Completely Randomized AOV under two factor factorial design for *W-usp-2***

**Source DF SS MS F P**

replication 1 0.00360 0.00360

Gene 2 3.556E-04 1.778E-04 2.5E+29 0.0000

Time 5 1.12926 0.22585 3.2E+32 0.0000

Gene*Time 10 0.00178 1.778E-04 2.5E+29 0.0000

Error 17 1.216E-32 7.155E-34

Total 35 1.13499

Grand Mean 0.3494 CV 3.96

Standard Error of a Mean 5.644E-03

**Completely Randomized AOV under two factor factorial design for mutant genes**

**Source DF SS MS F P**

replication 1 0.00360 0.00360

Gene 2 0.04887 0.02443 5.3E+32 0.0000

Time 5 0.85720 0.17144 3.7E+33 0.0000

Gene*Time 10 0.03233 0.00323 7.1E+31 0.0000

Error 17 7.794E-34 4.585E-35

Total 35 0.94200

Grand Mean 0.3033 CV 17.53

Standard Error of a Mean 0.0217

**LSD All-Pairwise Comparisons Test of Cell control**

**Gene Mean Homogeneous Groups**

1 0.4023 A

2 0.3950 B

3 0.3230 C

Alpha 0.05 Standard Error for Comparison 4.320E-03

**LSD All-Pairwise Comparisons Test of Cell by Time**

**Time Mean Homogeneous Groups**

16 0.4500 A

18 0.4000 B

12 0.3900 B

10 0.2900 C

8 0.2000 D

4 0.0900 E

Alpha 0.05 Standard Error for Comparison 7.303E-03

**LSD All-Pairwise Comparisons Test of Vector control**

**Gene Mean Homogeneous Groups**

1 0.5253 A

3 0.4363 B

2 0.4034 C

Alpha 0.05 Standard Error for Comparison 0.0324

**LSD All-Pairwise Comparisons Test of Vector by Time**

**Time Mean Homogeneous Groups**

16 0.5400 A

12 0.4533 B

18 0.4200 C

10 0.3367 D

8 0.1800 E

4 0.0800 F

Alpha 0.05 Standard Error for Comparison 8.344E-03

**LSD All-Pairwise Comparisons Test of *W-usp-2***

**Gene Mean Homogeneous Groups**

1 0.5237 A

3 0.4457 B

2 0.4320 C

Alpha 0.05 Standard Error for Comparison 0.0013

**LSD All-Pairwise Comparisons Test of *W-usp-2* by Time**

**Time Mean Homogeneous Groups**

16 0.5867 A

12 0.4900 B

18 0.4500 C

10 0.3200 D

8 0.1400 E

4 0.1100 F

Alpha 0.05 Standard Error for Comparison 7.981E-03

**LSD All-Pairwise Comparisons Test of mutant genes**

**Gene Mean Homogeneous Groups**

1 0.5102 A

3 0.4234 B

2 0.3755 C

Alpha 0.05 Standard Error for Comparison 7.320E-03

**LSD All-Pairwise Comparisons Test of mutant genes by Time**

**Time Mean Homogeneous Groups**

16 0.4500 A

12 0.4467 A

18 0.4400 A

10 0.2767 B

8 0.1300 C

4 0.0767 C

Alpha 0.05 Standard Error for Comparison 0.0307

**Table S7: List of Primers designed (by using Snap Gene software and Gene-Art primer) for Cloning of mutated and wild type *GUSP-2* genes in pCR2.1-TOPO, pET-30b and pCAMBIA-1301b expression vectors by using enzymes EcoRI + BamHI for pET-30b and BglII+ BstEII and SalI for pCAMBIA-1301b. Restriction sites are underlined. Mutated nucleotides are highlighted.**

| **Primer ID** | **Primer Sequence** | **Enzyme Used** |
| --- | --- | --- |
| Fd-M1-USP-2 | 5’-ACCAGTGGCTCACGT*CCC*TCACTGCCATACTCC-3’ | Nill |
| Rv-M1-USP-2 | 5’-GGAGTATGGCAGTGAGGGACGTGAGCCACTGGT-3’ | Nill |
| Fd-M2-USP-2 | 5’-CTTAAATGGACAATC*AGC*AATCTGGTAGACAAA-3’ | Nill |
| Rv-M2-USP-2 | 5’-TTTGTCTACCAGATTGCTGATTGTCCATTTAAG-3’ | Nill |
| Fd-M3-USP-2 | 5’-TAATCTCATGACCA*ACA*TCCCTTAACGTGAG-3’ | Nill |
| Rv-M3-USP-2 | 5’-CTCACGTTAAGGGATGTTGGTCATGAGATTA-3’ | Nill |
| Fd-BamHI-E-Y | 5’-GGATCCATGACAAAAGATAGGCAAATC-3’ | BamHI |
| Rv-EcoRI-E-Y | 5’-GAATTCTTACCTTTGAATGGTGC-3’ | EcoRI |
| Fd-BglII-P | 5’-AGATCTATGACAAAAGATAGGC-3’ | BglII |
| Rv-SalI-P | 5’-GTCGACTTACCTTTGAATGGTGC-3’ | SalI |
| Fd-GFP | 5’-GGTTCAGCTTTCTTGTACAAAGTGG-3’ | Nil |
| Rv-GFP | 5’-TTACTTGTACAGCTCGTCCATGC-3’ | Nil |
| Fd-SalI-GFP | 5’-GTCGACGGTTCAGCTTTCTTGTAC-3’ | SalI |
| Rv-BstEII-GFP | 5’-AGATCTTTACTTGTACAGCTCGTCCAT-3’ | BstEII |
| Fd-USP+GFP-S | 5'-ATGACAAAAGATAGGCAAATC-3’ | Nil |
| Re-USP+GFP-S | 5’-TTACTTGTACAGCTCGTCCAT-3’ | Nil |
| Fd-GAPDH | 5’-TGGGGCTACTCTCAAAGGGTTG-3’ | Nill |
| Fd-GAPDH | 5’-TGAGAAATTGCTGAAGCCGAAA-3’ | Nill |

**Table S8: Strains and plasmids used for cloning and expression study**

| Strain or Plasmids | Description |
| --- | --- |
| Strain |  |
| *E. coli* *BL-*21 | wild type |
| *E. coli* *BL-*21 | *uspA* mutant |
| *E. coli* *BL-*21 | *uspB* mutant |
| *E. coli* *BL-*21 | *uspC* mutant |
| *E. coli* *BL-*21 | *uspABC* mutant |
| *Pichia pastoris gs-115* | wild type |
| *E. coli* *TOP10* | wild type |
| Plasmids |  |
| pCR2.1 TOPO | Cat# K2020-20 |
| pET-30b | Cat # 69910-3, Merck Millipore |

**
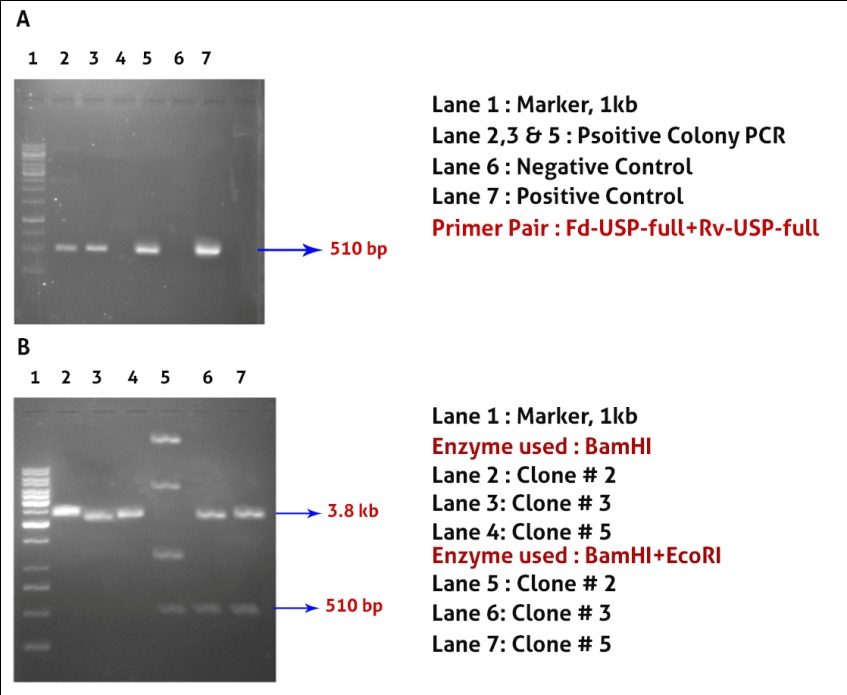
**

Figure S1: (A) PCR amplification & (B) Restriction digestion analysis


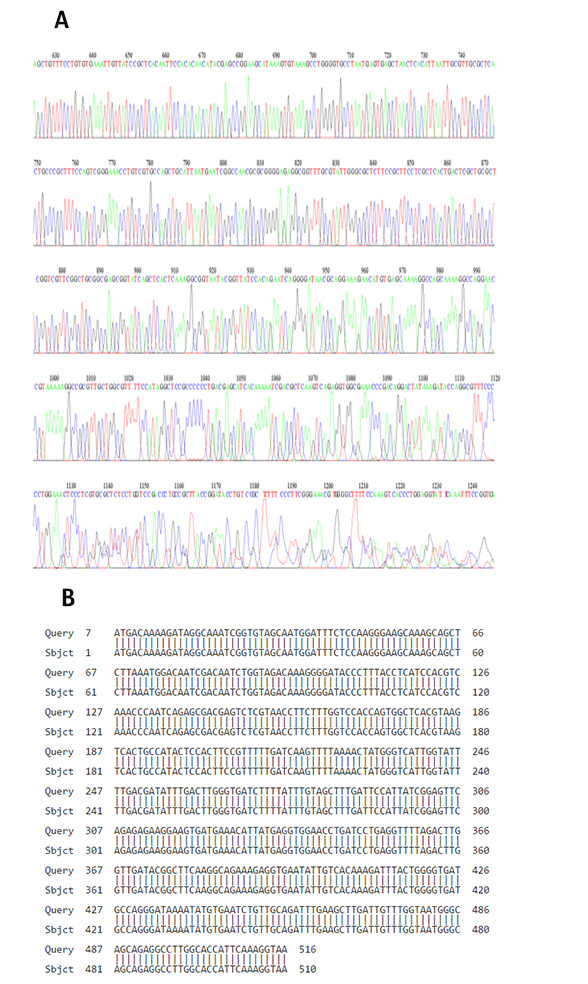


Figure S2: (A) Sequencing Peaks (<https://nucleobytes.com/4peaks/>) of *GUSP-2*, (B) BLAST of GUSP-2 with acc# EU107767


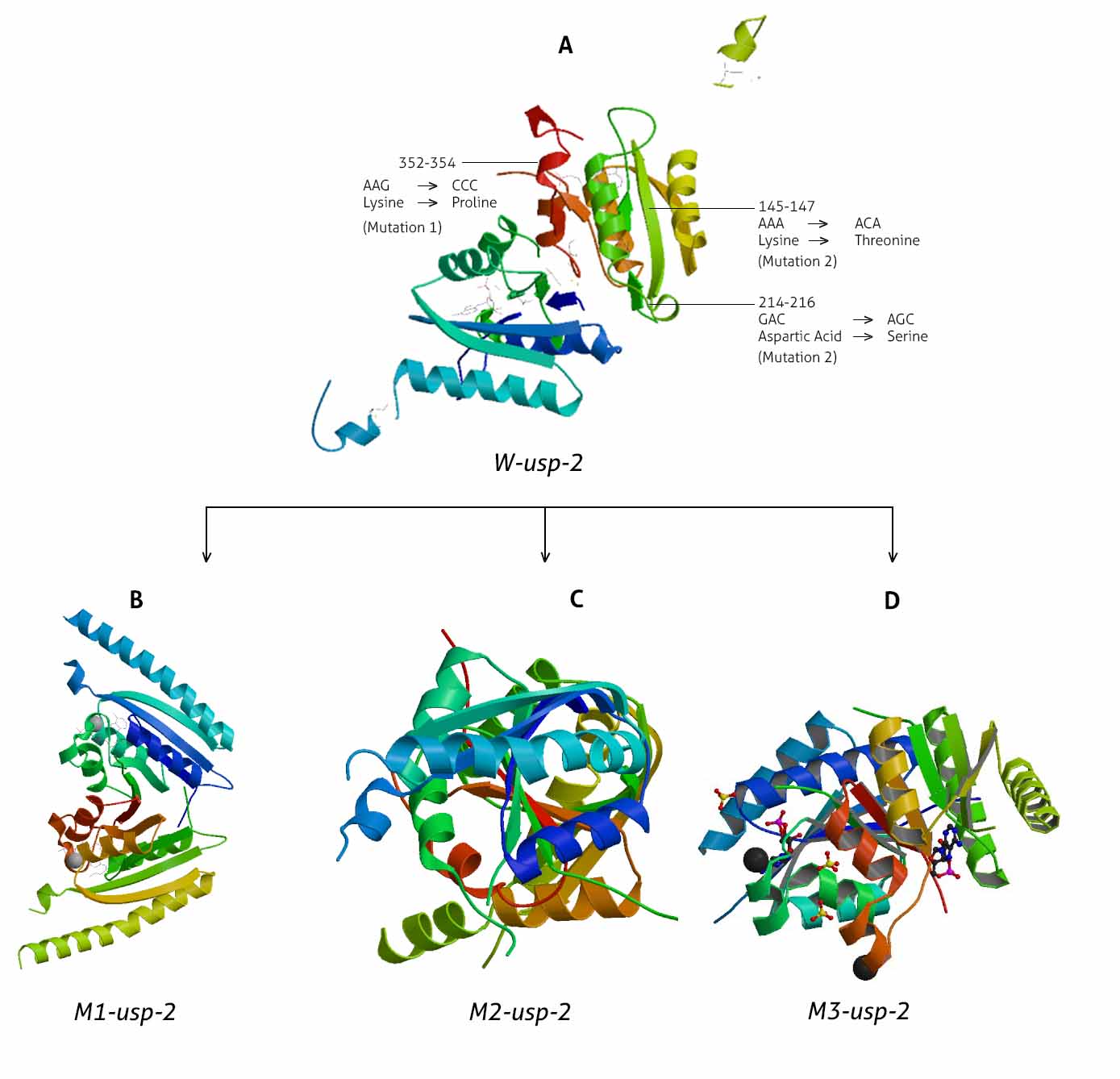


**Figure S3: Wild type GUSP-2 (A) has 28.23% identity with 2gm3.3 B unknown protein having 2XATP binding capacity, M1-usp-2 (B) mutated at 352-354 position (AAG replaced with CCC, Lycine replaced with Proline), it has 19.01% identity with 3ab8.1A putative uncharacterized protein TTHA0350 having 4XATP binding capacity, M2-usp-2 (C) mutated at 214-216 position (GAC replaced with AGC, Aspartic Acid replaced with Serine), it has 20.16% identity with 2dum.1.A hypothetical protein PH0823 having zero ATP binding capacity, (D) M3-usp-2 mutated model (mutated at 145-147 position, AAA replaced with CAC, Lysine to Threonine) has 21.49% identity with 5ahw.1.B Universal Stress Protein having 4XCMP binding capacity.**

MOE software (<https://www.chemcomp.com/Products.htm>) was used for the identification of ATP-binding sites in template protein structure which interacted with ATP


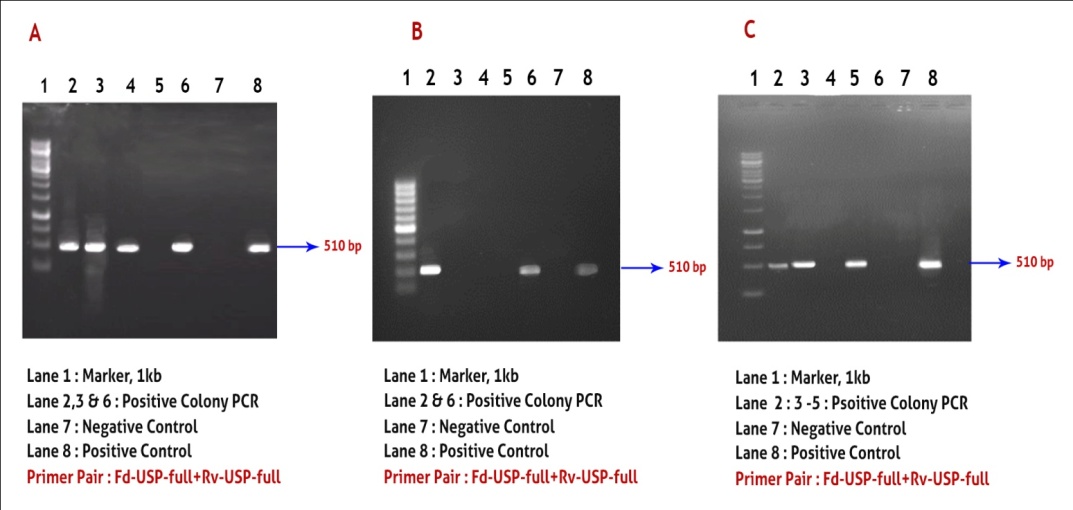


Figure S4: Colony PCR of (A) *Mp1,* (B) *Mp2 &* (C) *Mp3*

*
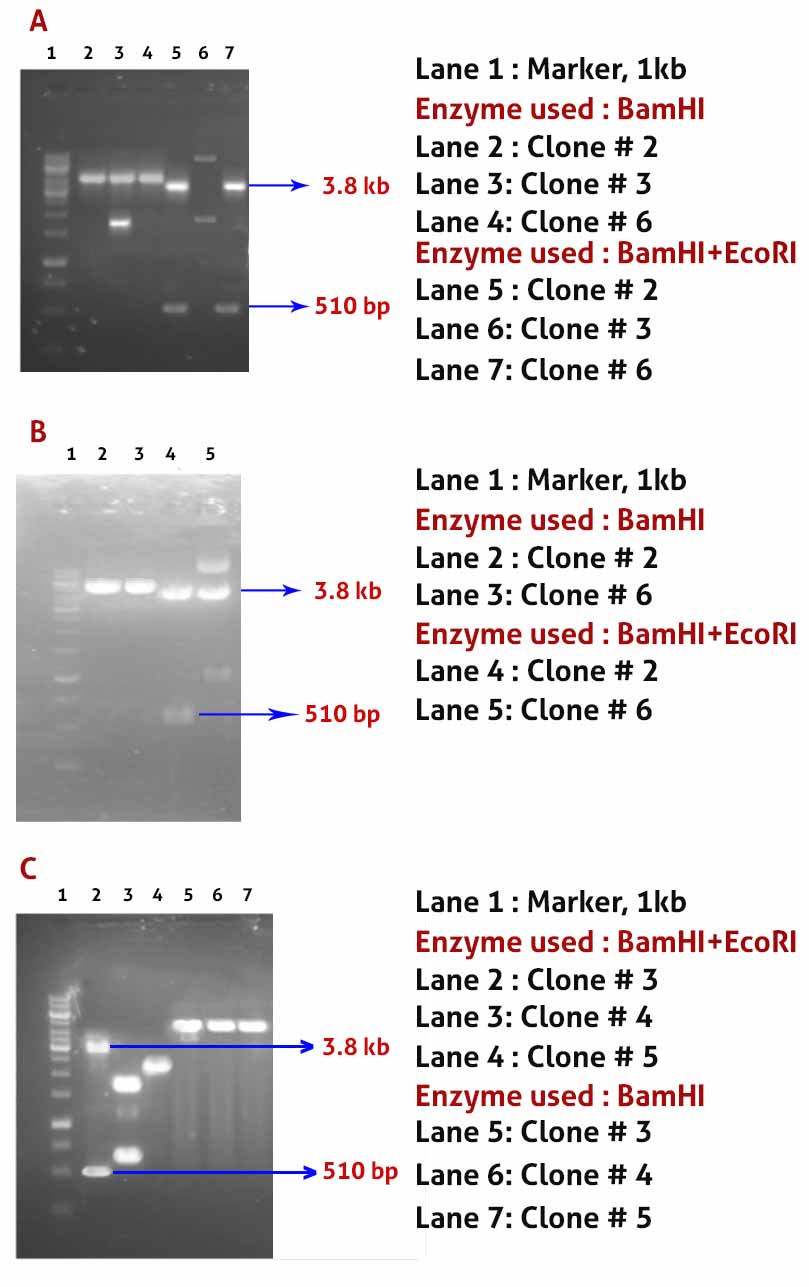
*

Figure S5: Restriction digestion analysis of (A) *Mp1,* (B) *Mp2* & (C) *Mp3*


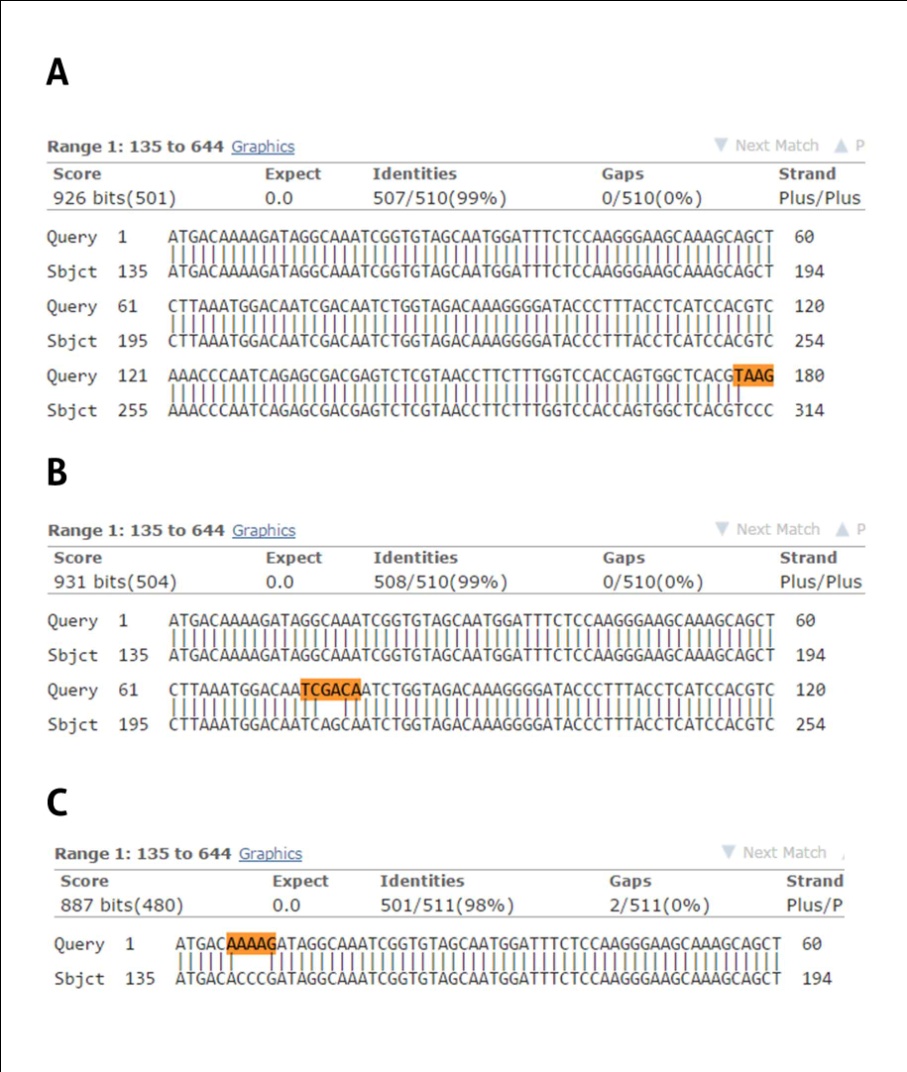


Figure S6: BLAST (<https://ncbi.github.io/magicblast>) of *GUSP-2* with (A) *M1-usp-2,* (B) *M2-usp-2* & (C) *M3-usp-2*

**
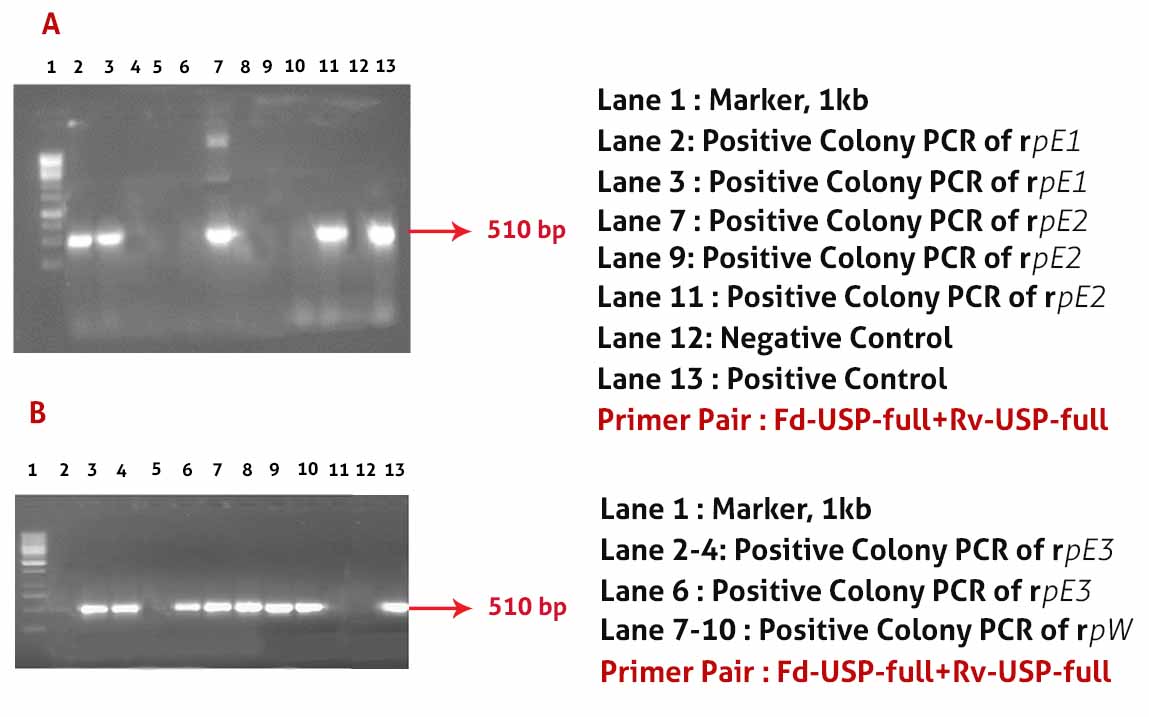
**

Figure S7: Colony P CR of (A) r*pE1*, (A) r*pE2*, (B) r*pE3* & (B) r*pEw*


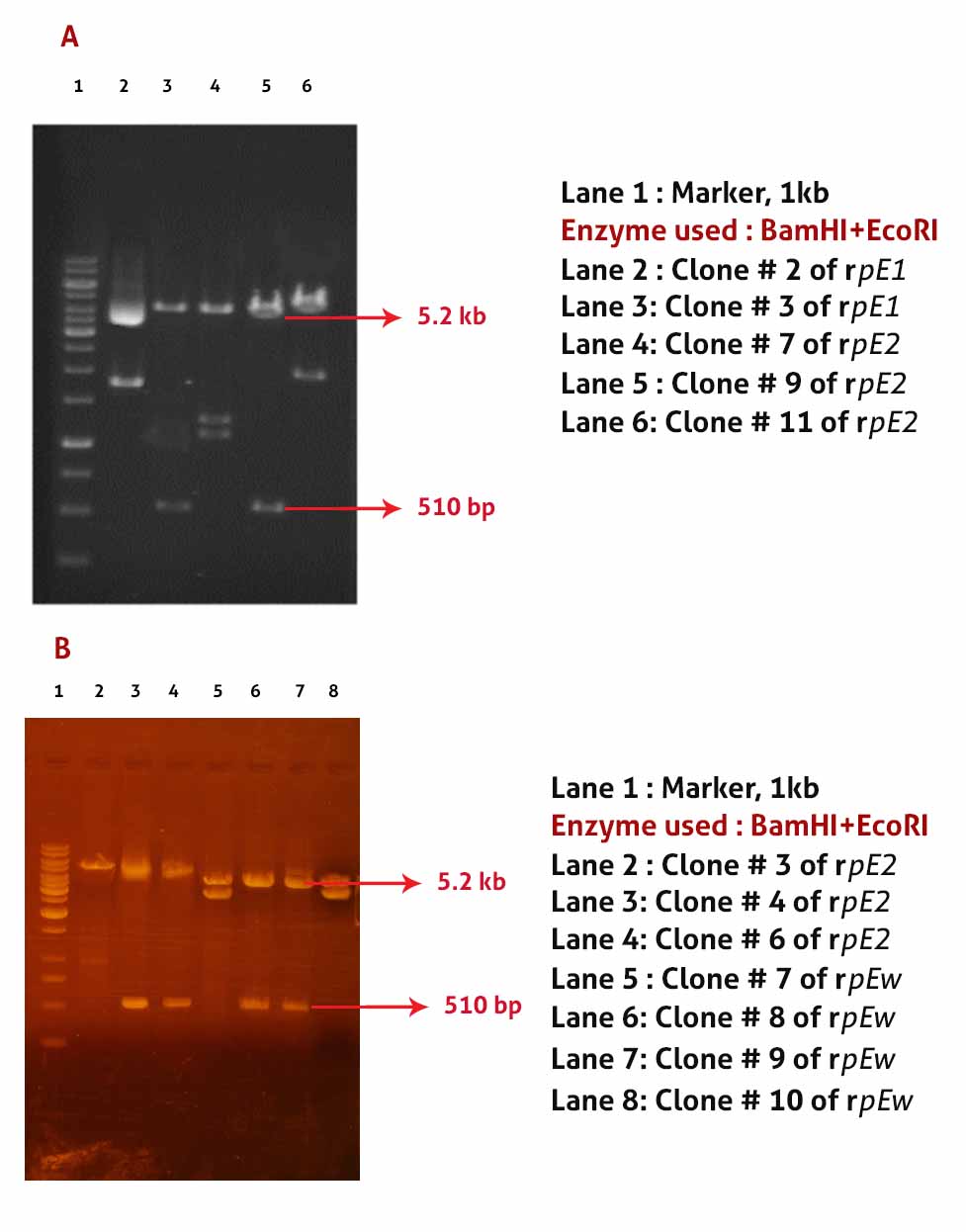


Figure S8: Restriction digestion analysis of (A) r*pE1*, (A) r*pE2*, (B) r*pE3* & (B) r*pEw*


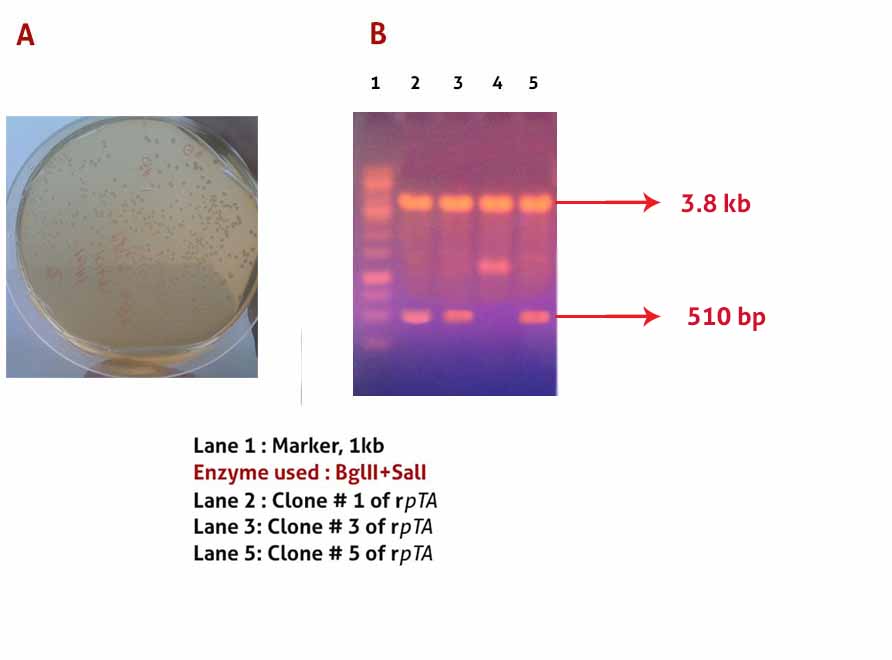


Figure S9: (A) Positive colonies of *TOP10 E.coli* transformed with recombinant pC2.1 vector, (B) Restriction digestion analysis of AT-clones


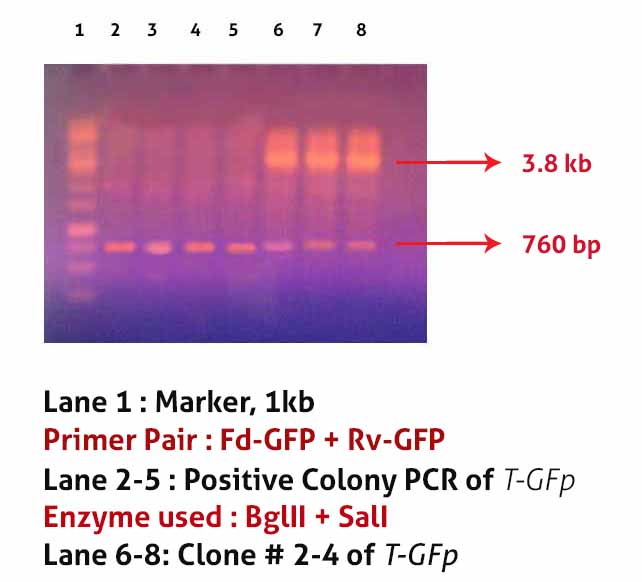


Figure S10: Colony PCR of *GFP* from T-*GFp* & Restriction digestion analysis of T-*GFp*


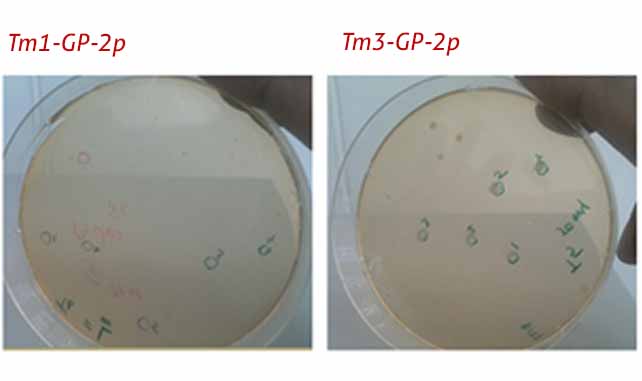


Figure S11: Positive colonies of *TOP10 E.coli* transformed with mutated pC2.1 vector


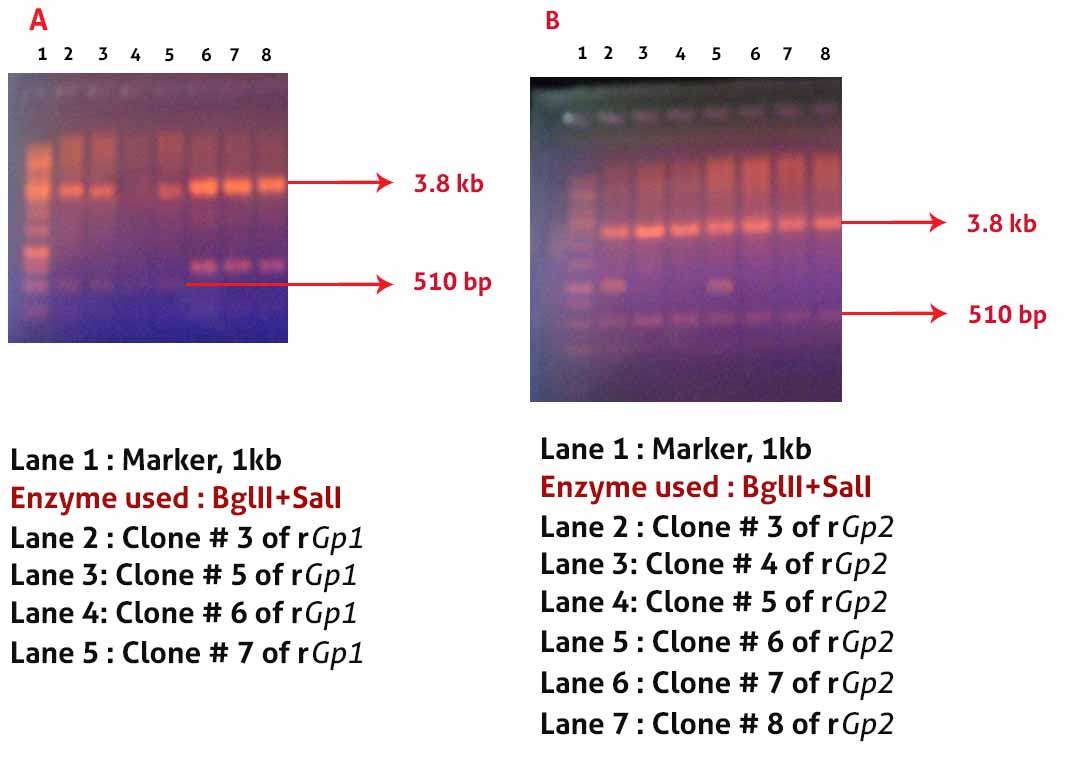


Figure S12: Restriction digestion analysis of mutated TA-clones for pCAMBIA-1301b


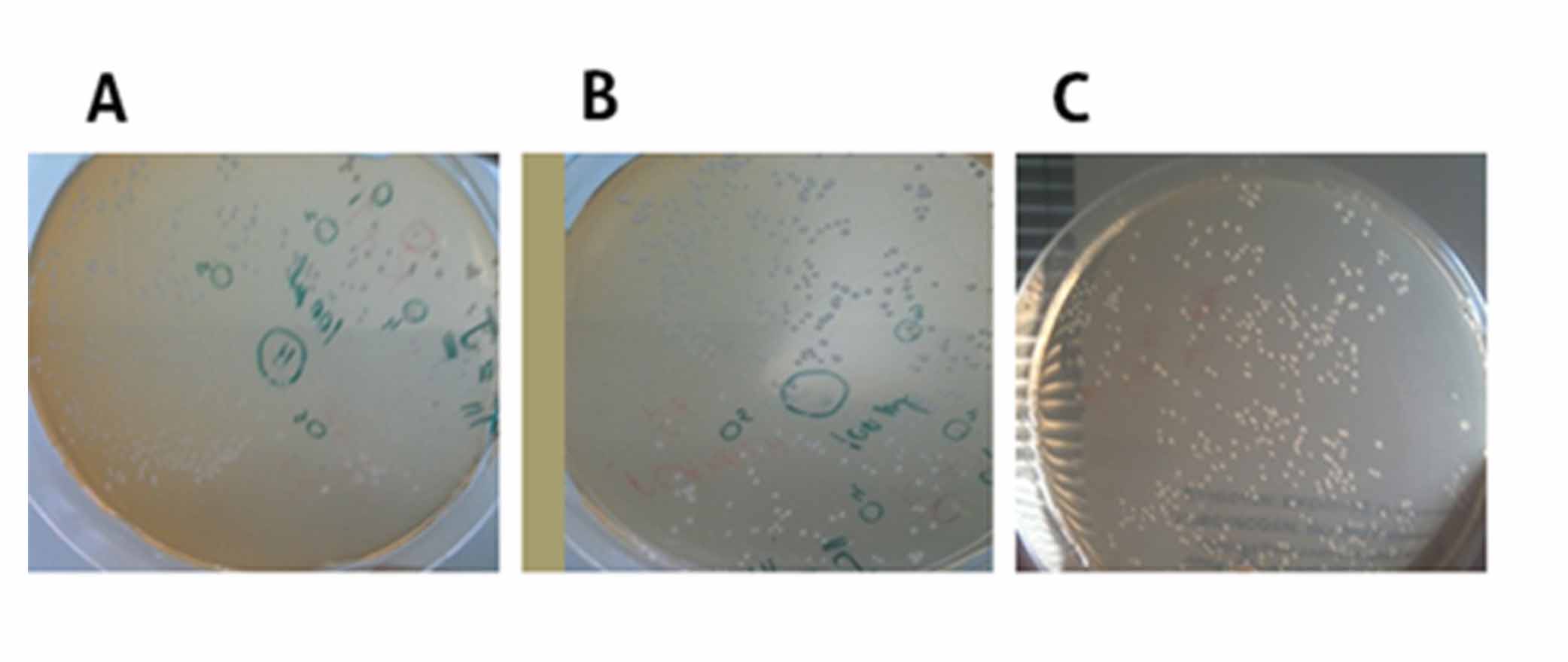


Figure S13: Transformed colonies of *Agrobacterium tumefaciens- LBA-4404* with p*W-usp-2* (A), p*M1-usp-2* (B) and p*GM3-usp-2* (C)


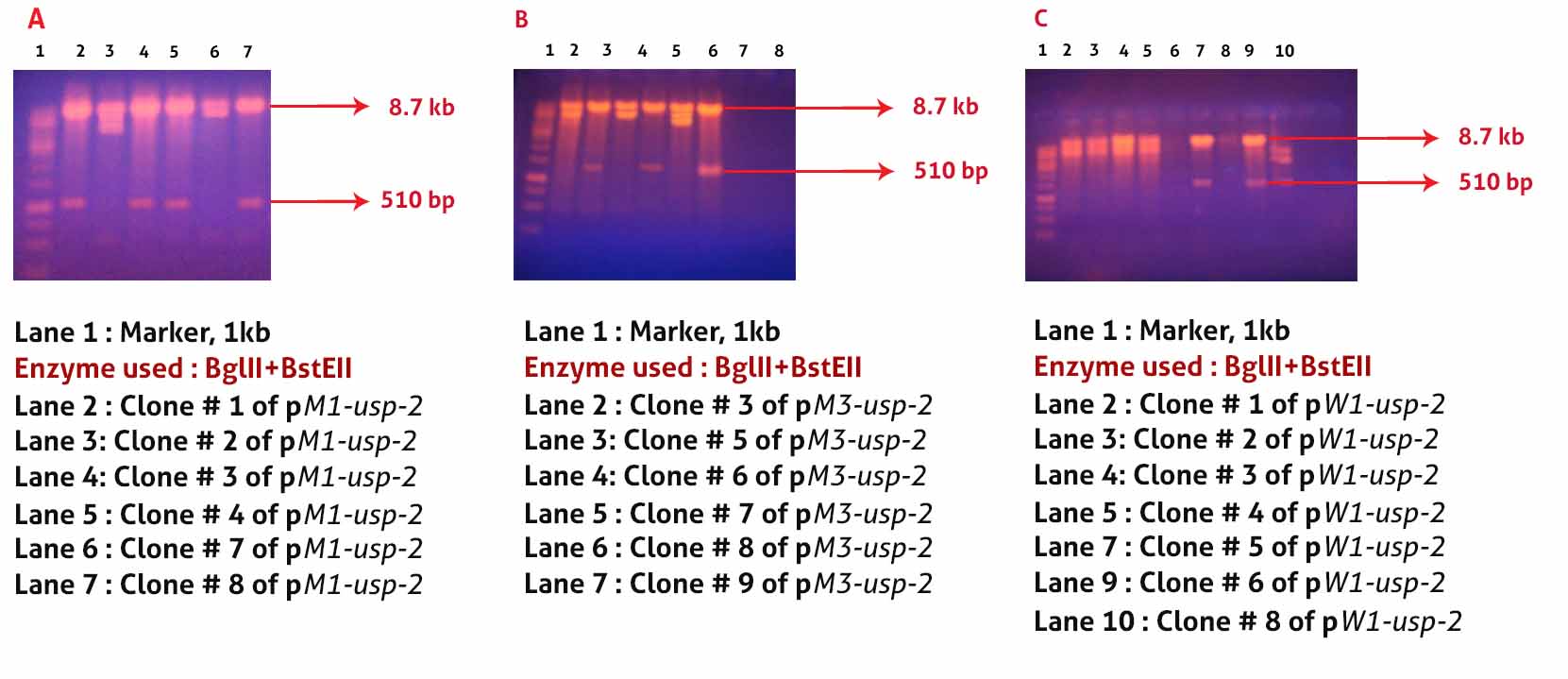


Figure S14: Restriction digestion analysis of positive transformants of p*W-usp-2* (A), p*M1-usp-2* (B) and p*M3-usp-2* (C)

**Table S9. Phenotypic data for plant traits**

| **Replication** | **Stress** | **Genes** | **Plant height** | **Shoot length** | **root length** | **Root shoot ratio** |
| --- | --- | --- | --- | --- | --- | --- |
| 1 | 1 | pT-W-usp-2 | 16.1 | 16.1 | 6.2 | 0.36 |
| 1 | 1 | pT-M1-usp-2 | 14.7 | 14.7 | 5.5 | 0.37 |
| 1 | 1 | pT-M3-usp-2 | 15.3 | 15.3 | 5.7 | 0.37 |
| 1 | 1 | control plants | 15.6 | 15.6 | 6 | 0.38 |
| 1 | 2 | pT-W-usp-2 | 25.2 | 25.2 | 11.6 | 0.46 |
| 1 | 2 | pT-M1-usp-2 | 24.3 | 24.3 | 12.8 | 0.52 |
| 1 | 2 | pT-M3-usp-2 | 24.8 | 24.8 | 12 | 0.48 |
| 1 | 2 | control plants | 24.8 | 24.8 | 10.9 | 0.43 |
| 1 | 3 | pT-W-usp-2 | 21.9 | 21.9 | 8.7 | 0.38 |
| 1 | 3 | pT-M1-usp-2 | 23.4 | 23.4 | 10.1 | 0.43 |
| 1 | 3 | pT-M3-usp-2 | 22.7 | 22.7 | 9 | 0.39 |
| 1 | 3 | control plants | 17.2 | 17.2 | 6.9 | 0.41 |
| 2 | 1 | pT-W-usp-2 | 16.44 | 16.44 | 6.54 | 0.394 |
| 2 | 1 | pT-M1-usp-2 | 15.04 | 15.04 | 5.84 | 0.404 |
| 2 | 1 | pT-M3-usp-2 | 15.64 | 15.64 | 6.04 | 0.404 |
| 2 | 1 | control plants | 15.94 | 15.94 | 6.34 | 0.414 |
| 2 | 2 | pT-W-usp-2 | 25.54 | 25.54 | 11.94 | 0.494 |
| 2 | 2 | pT-M1-usp-2 | 24.64 | 24.64 | 13.14 | 0.554 |
| 2 | 2 | pT-M3-usp-2 | 25.14 | 25.14 | 12.34 | 0.514 |
| 2 | 2 | control plants | 25.14 | 25.14 | 11.24 | 0.464 |
| 2 | 3 | pT-W-usp-2 | 22.24 | 22.24 | 9.04 | 0.414 |
| 2 | 3 | pT-M1-usp-2 | 23.74 | 23.74 | 10.44 | 0.464 |
| 2 | 3 | pT-M3-usp-2 | 23.04 | 23.04 | 9.34 | 0.424 |
| 2 | 3 | control plants | 17.54 | 17.54 | 7.24 | 0.444 |
| 3 | 1 | pT-W-usp-2 | 16.65 | 16.65 | 6.75 | 0.415 |
| 3 | 1 | pT-M1-usp-2 | 15.25 | 15.25 | 6.05 | 0.425 |
| 3 | 1 | pT-M3-usp-2 | 15.85 | 15.85 | 6.25 | 0.425 |
| 3 | 1 | control plants | 16.15 | 16.15 | 6.55 | 0.435 |
| 3 | 2 | pT-W-usp-2 | 25.75 | 25.75 | 12.15 | 0.515 |
| 3 | 2 | pT-M1-usp-2 | 24.85 | 24.85 | 13.35 | 0.575 |
| 3 | 2 | pT-M3-usp-2 | 25.35 | 25.35 | 12.55 | 0.535 |
| 3 | 2 | control plants | 25.35 | 25.35 | 11.45 | 0.485 |
| 3 | 3 | pT-W-usp-2 | 22.45 | 22.45 | 9.25 | 0.435 |
| 3 | 3 | pT-M1-usp-2 | 23.95 | 23.95 | 10.65 | 0.485 |
| 3 | 3 | pT-M3-usp-2 | 23.25 | 23.25 | 9.55 | 0.445 |
| 3 | 3 | control plants | 17.75 | 17.75 | 7.45 | 0.465 |
